# Synthesis and Anti-Tuberculosis Activity of the Marine Natural Product Caulerpin and Its Analogues

**DOI:** 10.3390/md12041757

**Published:** 2014-03-27

**Authors:** Cristina I. Canché Chay, Rocío Gómez Cansino, Clara I. Espitia Pinzón, Rubén O. Torres-Ochoa, Roberto Martínez

**Affiliations:** 1 Instituto de Química, Universidad Nacional Autónoma de México, Circuito Exterior, Ciudad Universitaria, Delegación Coyoacán, México D.F. C.P. 04510, Mexico; E-Mails: qfbcicc@hotmail.com (C.I.C.C.); chio746_@hotmail.com (R.G.C.); rube_to83@hotmail.com (R.O.T.-O.); 2 Instituto de Investigaciones Biomédicas, Universidad Nacional Autónoma de México, Ciudad Universitaria, Delegación Coyoacán, México D.F. C.P. 04510, Mexico.; E-Mail: espitia@biomedicas.unam.mx

**Keywords:** synthesis, caulerpin, analogues, anti-tuberculosis activity

## Abstract

Caulerpin (**1a**), a bis-indole alkaloid from the marine algal *Caulerpa* sp., was synthesized in three reaction steps with an overall yield of 11%. The caulerpin analogues (**1b**–**1g**) were prepared using the same synthetic pathway with overall yields between 3% and 8%. The key reaction involved a radical oxidative aromatic substitution involving xanthate (**3**) and 3-formylindole compounds (**4a**–**4g**). All bis-indole compounds synthesized were evaluated against the *Mycobacterium tuberculosis* strain H37Rv, and **1a** was found to display excellent activity (IC_50_ 0.24 µM).

## 1. Introduction

Tuberculosis (TB), caused mainly by the bacillus *Mycobacterium tuberculosis*, is the second cause of death worldwide due to an infectious disease, after human immunodeficiency virus (HIV/AIDS). In 2012, the World Health Organization (WHO, Geneva, Switzerland) reported almost nine million new cases of TB, 1.3 million deaths due to TB and 0.3 million deaths resulting from a co-infection with HIV and TB [1].

Natural products are sources of active compounds that may be useful in the development of new drugs. Bis-indole-based alkaloids are an important family of alkaloid compounds that are widespread in nature and display diverse and interesting biological activities as anti-tumor or antibacterial agents, showing particular activity against different species of Mycobacteria. Some of these compounds have been obtained from marine natural products [2,3,4,5,6]. The importance of marine metabolites may be appreciated in view of the fact that three approved drugs for the U.S. FDA (Food and Drug Administration, Silver Spring, MD, USA) have been isolated from marine species, and thirteen additional natural compounds (or their synthetic derivatives) are in different phases of clinical trials [7].

Caulerpin (**1a**) is a bis-indole alkaloid isolated from *Caulerpa racemosa* and *C. serrulata* in 1970 in a yield of 0.55%–0.63% the algal dry weight [8]. Only one synthetic route to **1a** has been reported from the 3-formylindol-2-yl acetic ester, providing a 5% yield [9]. In 1984, it was described that the compound had a low toxicity [10] and a variety of biological activities have been reported [11,12,13,14,15,16,17,18].

Despite showing a wide range of promising biological activities, the only source of this compound has been the marine alga, *Caulerpa* sp. Thus far, no accessible synthetic methods have been described for obtaining **1a** in good yields. With these considerations in mind, the present investigation was undertaken to develop an efficient method for synthesizing **1a** and its analogues (**1b**–**1g**). The activities of these compounds against the *M. tuberculosis* strain, H37Rv, were evaluated.

## 2. Results and Discussion

### 2.1. Chemistry

**Scheme 1:**
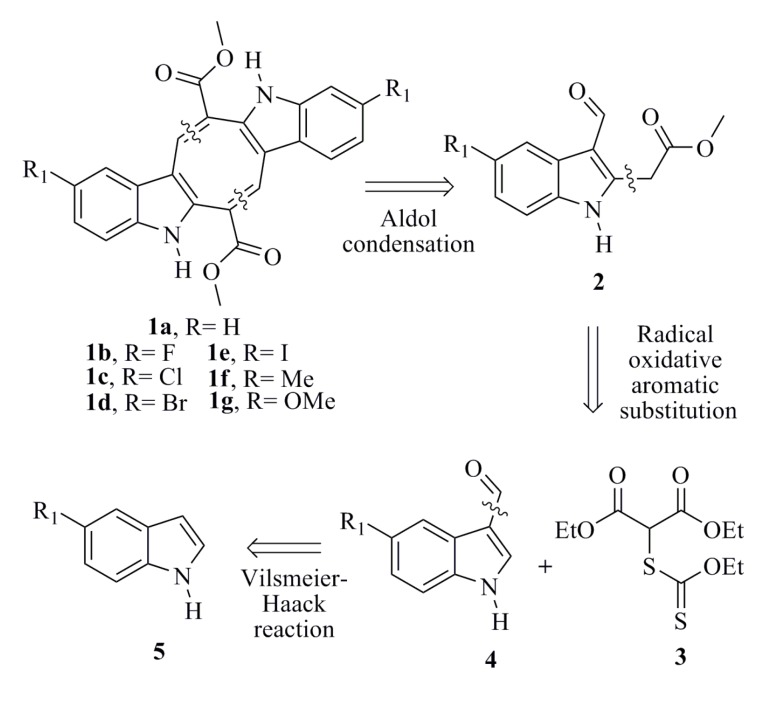
Retrosynthetic analysis of **1a** and its analogues (**1b**–**1g**).

Since **1a** and its analogues (**1b**–**1g**) possessed C-2 symmetry, their synthesis could be achieved through an aldol condensation from the ester derivatives (**2**). The indoles **2** could be formed via a radical oxidative aromatic substitution reaction between the 3-formylindoles (**4**) and xanthate (**3**). Finally, the indoles-3-carboxaldehydes could be obtained from the indole through a Vilsmeier-Haack reaction (**5**) (Scheme 1).

**Scheme 2:**
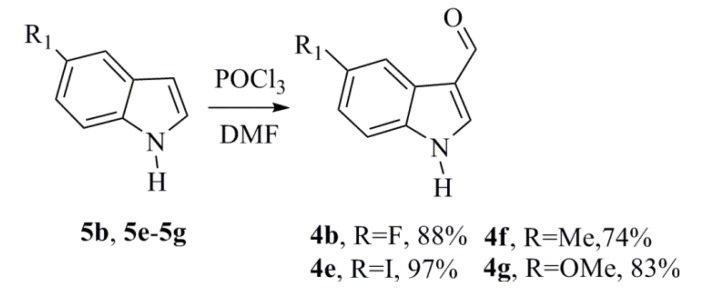
Synthesis of the 3-formylindoles **4b**, **4e**–**4g**.

The synthesis of **1a**–**1g** started with the formylation reaction of the 5-substituted-indole (**5b**, **5e**–**5g**) using POCl_3_ and DMF in good to excellent yields (Scheme 2) [19]. This reaction proceeded due to the preference of the electrophilic aromatic substitution reaction for attack at C3 on the indole. The aldehydes **4a**, **4c**, and **4d** were acquired commercially.

**Scheme 3:**
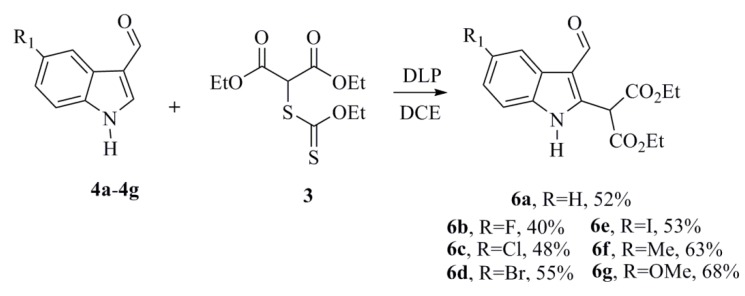
Reaction conditions for the synthesis of **6a**–**6g**.

Few synthetic methods available today can provide the 2-substituted indoles. Some of the available methodologies rely on ionic reactions to access this type of indole, and few methods involve free radicals. In 2003, Miranda *et al.* [20] reported the synthesis of heteroaromatic compounds via a radical aromatic substitution reaction that used various dithiocarbonates (xanthates) and dilauroyl peroxide (DLP) as the initiator and oxidizing agent, respectively. Based on these reports, we decided to synthesize the malonic derivatives (**6a**–**6g**) using xanthate **3** and the indoles 3-carboxaldehydes (**4a**–**4g**) by adjusting the amounts of these compounds until optimal conditions were identified to obtain the best yields; however, all products included the recovered starting material (**4a**–**4g**). It should be noted that xanthate **3** was synthesized by nucleophilic substitution between diethyl chloromalonate and potassium ethyl xanthogenate in a 97% yield. All the same, we were able to synthesize seven ester malonic derivatives in moderate yields (Scheme 3). It is important to mention that the preparation of the compounds **6a**–**6g** was accomplished, regardless of the presence of a protecting group on the amine moiety. These yields were attributed to the low nucleophilicity of the radical at C3 formed during this reaction. Moreover, in the case of **6e**, a mixture of 1,2-dichloroethane (DCE) and toluene was used, due to the low solubility of the indol-3-carboxaldehyde in DCE (Scheme 3).

**Scheme 4:**
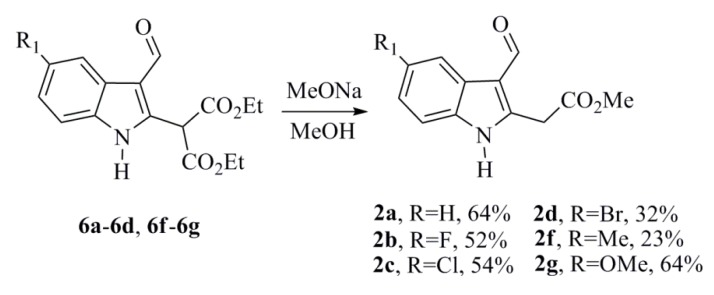
Optimal reaction conditions for the synthesis of **2a**–**2d** and **2f**–**2g**.

The subsequent decarboxylation and transesterification reactions of the indoles **6a**–**6d** and **6f**–**6g** with MeOH and MeONa afforded compounds **2a**–**2d** and **2f**–**2g** in low to moderate yields, possibly due to the indirect hydrolysis of the malonic ester (Scheme 4) [21].

**Scheme 5:**
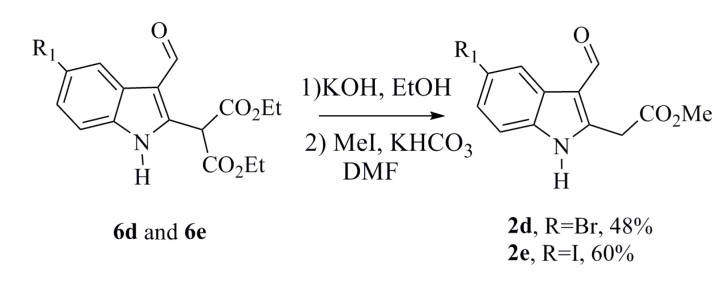
Alternative synthesis of the indoles **2d** and **2e**.

The indoles **2d** and **2e** were obtained from the decarboxylation and transesterification step in low yields. In an effort to increase the yields, the hydrolysis reactions were conducted using KOH, and the alkylation reaction was performed using MeI. The desired compounds **2d** and **2e** were thereby synthesized in an optimal manner (Scheme 5) [22,23].

Once the ester **2a** had been prepared, we focused our attention on the synthesis of the natural product **1a** based on the reaction conditions that allowed Maiti *et al.* [9] to achieve the first synthesis of the natural product. For this purpose, several experiments were carried out in which certain factors were modified, such as the solvent, the amount of piperidine and diethylamine, and the concentration of the substrate. Temperature was thought to play an important role in the transformation, and high temperatures favored the condensation and dehydration steps. For this reason, xylene and toluene were tested as solvents. The results improved for the reaction conducted with piperidine (0.023 M) and diethylamine (0.023 M): the yield increased to 32% and the reaction time decreased to only 3 h. The preparation of **1a** was corroborated by comparing the spectroscopic data of the synthetic and isolated products. No changes in the yield were detected, and the reaction was achieved at higher concentrations, 0.038 or 0.23 M. The highest substrate concentration produced a considerable decrease in the yield due to the formation of many by-products, thereby supporting the importance of the dilution factor. Unexpectedly, the use of toluene provided a lower yield. It is important to note that the best dimerization yield was low (32%), although remarkably better than the results obtained previously (see Supplementary Information).

**Scheme 6:**
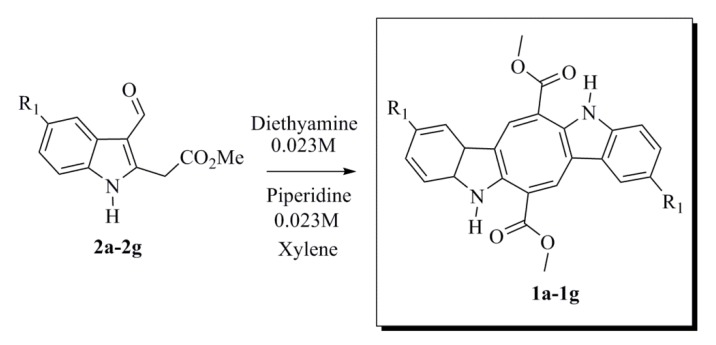
Reaction conditions for the synthesis of **1a**–**1g**.

Our next task was to employ the best reaction conditions in the preparation of the caulerpin analogues (**1b**–**1g**). The six novel compounds were obtained in similar yields, except that the chloro and iodo analogues (**1c**, **1e**) provided low yields. During the syntheses of **1b** and **1g**, the starting materials were not consumed until the reaction had proceeded for 10 h (Scheme 6 and Table 1). All synthesized compounds were characterized using spectroscopic methods.

**Table 1:** Reaction conditions for the synthesis of **1a**–**1g**.

Compound	Substituent (R_1_)	Temperature (°C)	Time (h)	Yield (%)
**1a**	H	reflux	3	32
**1b**	F	reflux	10	24
**1c**	Cl	reflux	3	17
**1d**	Br	reflux	3	32
**1e**	I	reflux	5	10
**1f**	Me	reflux	3	24
**1g**	OMe	reflux	10	23

### 2.2. Anti-Tuberculosis Activity

**Figure 1:**
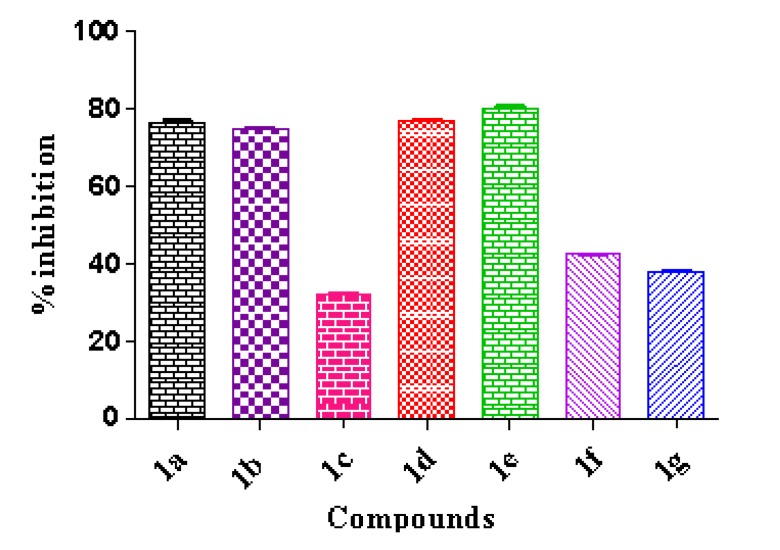
The percent of growth inhibition of the compounds **1a**–**1g** against the *M. tuberculosis* strain H37Rv. Values are the means ± S.E.M., *n* = 5. Assay concentration 50 µM.

**Figure 2:**
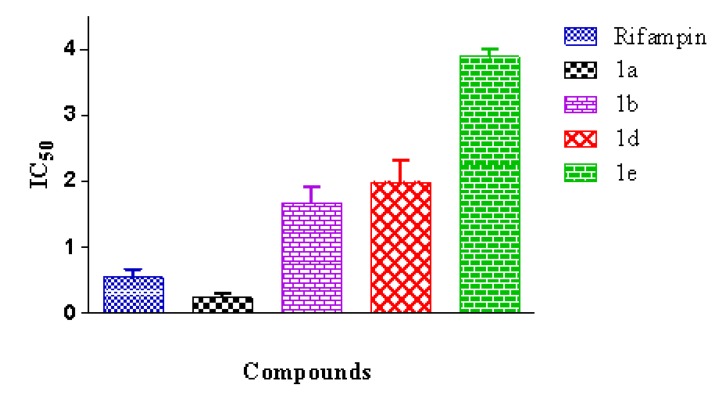
IC_50_ values of compounds **1a**, **1b**, **1d** and **1e** against the *M. tuberculosis* strain H37Rv. Values are the means ± S.E.M., *n* = 5.

We evaluated the activities of compounds **1a**–**1g** as inhibitors of the growth of the *Mycobacterium tuberculosis* strain, H37Rv, at a 50 µM concentration. Compounds **1a**, **1b**, **1d**, and **1e** showed a percentage of inhibition exceeding 70% (Figure 1). These compounds displayed IC_50_ values as low as 0.24 µM for caulerpin, and as high as 3.89 µM for **1e** (Figure 2). Rifampin (RIF) is usually indicated for the treatment of *Mycobacterium* infections, including tuberculosis, and the anti-tuberculosis activities of the synthesized compounds **1a**–**1g** were compared with the activity of rifampin. As shown in Figure 2, RIF (IC_50_ = 0.55 µM) was more active than the synthesized bis-indoles **1b** (IC_50_ = 1.68 µM), **1d** (IC_50_ = 1.98 µM), and **1e** (IC_50_ = 3.89 µM), whereas caulerpin **1a** showed a potency that was more than twice that of RIF (IC_50_ = 0.24 µM).

## 3. Experimental Section

### 3.1. Materials and Synthetic Procedure

Melting points were determined using a Melt-Temp II melting point apparatus and are uncorrected. IR spectra were recorded using a Nicolet FT Magna-IR 750 spectrometer (Thermo, Madison, WI, USA). The ^1^H-NMR spectra were recorded using the Gemini-200 MHz Varian (Varian, Palo Alto, CA, USA) and a Jeol Eclipse 300 (Jeol Ltd., Tokyo, Japan), and samples were prepared in deuterated chloroform solutions containing tetramethylsilane or deuterated dimethylsulfoxide. ^13^C-NMR spectra were recorded at 75 MHz on the same instruments. The peak patterns are indicated as follows: s, singlet; d, doublet; t, triplet; q, quartet; m, multiplet; bs, broad singlet. The coupling constants (*J*) are reported in Hertz (Hz). Mass spectra were obtained using a Jeol JMS-AX505HA and Jeol 5X102A mass spectrometers (Jeol Ltd.) by electronic impact (EI) and fast atom bombardment (FAB+) ionization. The purification of compounds by column chromatography was performed on Silica Gel 60 F254 Merck (Merck, Darmstadt, Gemany). Commercial-grade reagents were used with further purification.

#### 3.1.1. General Procedure for Synthesizing the Indoles-3-Carboxaldehydes (**4b** and **4e**–**4g**)

Phosphorus oxychloride (0.42 g, 2.74 mmol) was added dropwise to a solution of the indole **5b**, **5e**–**5g** (0.30 g, 2.29 mmol) in DMF (0.84 g, 11.4 mmol) at 0 °C for 30 min. The solution was then heated at 40 °C for 1 h. Ice was added to the reaction vessel, followed by a solution of sodium hydroxide (2 M). The solution was refluxed for 40 min. The mixture was cooled and extracted using ethyl acetate, and the organic phase was washed with brine. The organic extracts were combined, dried over Na_2_SO_4_, and concentrated. The crude residue was purified by chromatography on a silica gel column using hexane-ethyl acetate as an eluent to obtain the desired product [19].

The indole derivatives **4a**, **4c**, and **4d** were purchased from Sigma-Aldrich (Sigma-Aldrich, St. Louis, MO, USA).

5-Fluoro-1*H*-indole-3-carbaldehyde (**4b**)

Yellow solid; yield 88%; mp 158–160 °C; ^1^H-NMR (300 MHz, CDCl_3_ + DMSO-*d*_6_) *δ* 11.72 (bs, 1H), 9.96 (s, 1H), 7.92 (d, *J* = 3.0 Hz, 1H), 7.88 (dd, *J* = 9.4, 2.4 Hz, 1H), 7.42 (dd, *J* = 8.8, 4.2 Hz, 1H), 7.01 (dt, *J* = 9.0, 2.4 Hz, 1H) [24].

5-Iodo-1*H*-indole-3-carbaldehyde (**4e**)

Yellow solid; yield 97%; mp 192–194 °C; ^1^H-NMR (300 MHz, CDCl_3_ + DMSO-*d*_6_) *δ* 11.72 (s, 1H), 9.95 (bs, 1H), 8.59 (d, *J* = 1.8 Hz, 1H), 7.84 (d, *J* = 3.0 Hz, 1H), 7.53 (dd, *J* = 8.5, 1.8 Hz, 1H), 7.27 (d, *J* = 8.4 Hz, 1H) [25].

5-Methyl-1*H*-indole-3-carbaldehyde (**4f**)

Yellow solid; yield 74%; mp 144–146 °C; ^1^H-NMR (300 MHz, CDCl_3_) *δ* 10.02 (s, 1H), 9.21 (bs, 1H), 8.13 (s, 1H), 7.81 (d, *J* = 3.3 Hz, 1H), 7.34 (d, *J* = 8.4 Hz, 1H), 7.15 (d, *J* = 8.4 Hz, 1H), 2.47 (s, 3H) [26].

5-Methoxy-1*H*-indole-3-carbaldehyde (**4g**)

Light brown solid; yield 83%; mp 176–178 °C; ^1^H-NMR (200 MHz, CDCl_3_ + DMSO-*d*_6_) *δ* 12.08 (bs, 1H), 9.93 (s, 1H), 8.25 (s, 1H), 7.62 (d, *J* = 2.6 Hz, 1H), 7.44 (d, *J* = 8.8 Hz, 1H), 6.92 (dd, *J* = 8.8, 2.6 Hz, 1H),3.82 (s, 3H) [27].

#### 3.1.2. Procedure for Synthesizing the Xanthate of Diethyl Malonate (**3**)

To a solution of diethyl chloromalonate (1 g, 5.14 mmol) in acetonitrile (6.4 mL) was added potassium ethyl xanthogenate (0.99 g, 6.17 mmol) at 0 °C, and the mixture was stirred for 1 h. The crude product was then concentrated under reduced pressure, washed with brine, and extracted with CH_2_Cl_2_. The organic extracts were combined, dried over Na_2_SO_4_, and concentrated in vacuum. The crude residue was purified by chromatography on a silica gel column using hexane-ethyl acetate as the eluent to obtain the desired product.

Diethyl 2-(Ethoxycarbonothioylthio)malonate (**3**)

Yellow oil; yield 97%; ^1^H-NMR (300 MHz, CDCl_3_) *δ* 5.28 (s, 1H), 4.65 (q, *J* = 7.2 Hz, 2H), 4.27 (q, *J* = 7.2 Hz, 4H), 1.42 (t, *J* = 7.2 Hz, 3H), 1.30 (t, *J* = 7.2 Hz, 6H); ^13^C-NMR (75 MHz, CDCl_3_) *δ* 210.2, 165.1, 71.0, 62.8, 56.3, 13.9, 13.6 [28].

#### 3.1.3. General Procedure for Synthesizing the Diethyl 2-(3-formyl-1*H*-indol-2-yl)malonate Derivatives (**6a**–**6g**)

A solution of the xanthate **3** (1.37 g, 4.87 mmol) and the corresponding indole (**4a**–**4g**) (0.353 g, 2.21 mmol) in degassed 1,2-dichloroethane (DCE) (9 mL) was heated at reflux, and dilauroyl peroxide (DLP) (2.21 g, 5.54 mmol) in solid form was added in several portions (0.92 mmol/h). The reaction was monitored by TLC and was stopped after 6 h. The solvent was removed under reduced pressure, and the crude residues were extracted with acetonitrile and washed with hexane. The polar phase was then concentrated under reduced pressure and purified by chromatography on a silica gel column using hexane-ethyl acetate as the eluent to obtain the desired products.

Diethyl 2-(3-Formyl-1*H*-indol-2-yl)malonate (**6a**)

Yellow solid; yield 52%; mp 70–72 °C; IR (KBr) ν_max_ 3187, 1728, 1624 cm^−1^; ^1^H-NMR (300 MHz, CDCl_3_) *δ* 10.30 (s, 1H), 10.00 (bs, 1H), 8.20–8.17 (m, 1H), 7.46–7.26 (m, 3H), 5.74 (s, 1H), 4.35–4.19 (m, 4H), 1.29 (t, *J* = 7.2 Hz, 6H); ^13^C-NMR (75 MHz, CDCl_3_) *δ* 184.4, 166.3, 136.8, 135.3, 125.8, 124.2, 122.9, 120.2, 115.1, 111.8, 63.0, 49.0, 13.9; EIMS *m*/*z* 303 [M]^+^ (100), 157 (73).

Diethyl 2-(5-Fluoro-3-formyl-1*H*-indol-2-yl)malonate (**6b**)

Yellow solid; yield 40% (58% Based on Recovered Starting Material); mp 98–100 °C; IR (KBr) ν_max_ 3149, 3108, 2983, 2933, 1738, 1637 cm^−1^; ^1^H-NMR (300 MHz, CDCl_3_) *δ* 10.23 (s, 1H), 10.10 (bs, 1H), 7.86 (dd, *J* = 9.3, 2.7 Hz, 1H), 7.37 (dd, *J* = 8.9, 4.2 Hz, 1H), 7.05 (dt, *J* = 9.0, 2.7 Hz, 1H), 5.69 (s, 1H), 4.37–4.20 (m, 4H), 1.30 (t, *J* = 6.9 Hz, 6H); ^13^C-NMR (75 MHz, CDCl_3_) *δ* 184.0, 166.2, 159.7 (d, *J*_CF_ = 237.8 Hz, Ar-quat), 138.2, 131.7, 126.4 (d, *J*_CF_ = 10.5 Hz, Ar-quat), 115.3 (d, *J*_CF_ = 4.5 Hz, Ar-quat), 112.8 (d, *J*_CF_ = 5.3 Hz, Ar-CH), 112.6 (d, *J*_CF_ = 11.3 Hz, Ar-CH), 105.9 (d, *J*_CF_ = 25 Hz, Ar-CH), 63.1, 49.0, 13.9; EIMS *m*/*z* 321 [M]^+^ (37), 181 (70), 115 (100); HRMS (FAB+) *m*/*z* calcd. for C_16_H_17_O_5_NF, 322.1091; found: 322.1082.

Diethyl 2-(5-Chloro-3-formyl-1*H*-indol-2-yl)malonate (**6c**)

Yellow solid; yield 48% (69% Based on Recovered Starting Material); mp 102–104 °C; IR (KBr) ν_max_ 3139, 2979, 2826, 1734, 1633 cm^−1^; ^1^H-NMR (300 MHz, CDCl_3_) *δ* 10.24 (s, 1H), 10.00 (bs, 1H), 8.19 (d, *J* = 2.1 Hz, 1H), 7.36 (d, *J* = 8.7 Hz, 1H), 7.25 (dd, *J* = 8.7 Hz, 2.1 Hz, 1H), 5.68 (s, 1H), 4.37–4.20 (m, 4H), 1.31 (t, *J* = 7.2 Hz, 6H); ^13^C-NMR (75 MHz, CDCl_3_) *δ* 184.0, 166.1, 137.9, 133.6, 128.9, 126.7, 124.6, 120.1, 114.8, 112.8, 63.2, 48.9, 13.9; EIMS *m*/*z* 337 [M]^+^ (84), 338 (22), 339 (34), 340 (11); HRMS (FAB+) *m*/*z* calcd. for C_16_H_17_O_5_NCl, 338.0795; found: 338.0801.

Diethyl 2-(5-Bromo-3-formyl-1*H*-indol-2-yl)malonate (**6d**)

Yellow solid; yield 56%; mp 108–110 °C; IR (KBr) ν_max_ 3159, 2989, 1732, 1634 cm^−1^; ^1^H-NMR (300 MHz, CDCl_3_) *δ* 10.23 (s, 1H), 10.08 (bs, 1H), 8.35 (d, *J* = 1.8 Hz, 1H), 7.39 (dd, *J* = 8.7, 1.8 Hz, 1H), 7.31 (d, *J* = 8.7 Hz, 1H), 5.68 (s, 1H), 4.37–4.20 (m, 4H), 1.31 (t, *J* = 7.2 Hz, 6H); ^13^C-NMR (75 MHz, CDCl_3_) *δ* 184.0, 166.1, 137.8, 133.9, 127.3, 123.1, 116.5, 114.7, 113.2, 63.2, 48.9, 13.9; EIMS *m*/*z* 381 [M]^+^ (66), 383 (65); HRMS (FAB+) *m*/*z* calcd. for C_16_H_16_O_5_NBr, 381.0212; found: 381.0213.

Diethyl 2-(3-Formyl-5-iodo-1*H*-indol-2-yl)malonate (**6e**)

Yellow solid; yield 53%; mp 90–92 °C; IR (KBr) ν_max_ 3166, 2977, 2929, 1732, 1629 cm^−1^; ^1^H-NMR (300 MHz, CDCl_3_) *δ* 10.22 (s, 1H), 10.06 (bs, 1H), 8.56 (d, *J* = 1.5 Hz, 1H), 7.57 (dd, *J* = 8.5, 1.5 Hz, 1H), 7.22 (d, *J* = 8.4 Hz, 1H), 5.67 (s, 1H), 4.37–4.20 (m, 4H), 1.31 (t, *J* = 6.9 Hz, 6H); ^13^C-NMR (75 MHz, CDCl_3_) *δ* 184.0, 166.1, 137.4, 134.4, 132.8, 129.3, 127.9, 114.4, 113.6, 87.0, 63.2, 48.9, 13.9; EIMS *m*/*z* 429 [M]^+^ (100), 383 (87); HRMS (FAB+) *m*/*z* calcd. for C_16_H_17_O_5_NI, 430.0151; found: 430.0163.

Diethyl 2-(3-Formyl-5-methyl-1*H*-indol-2-yl)malonate (**6f**)

Yellow solid; yield 63%; mp 84–86 °C; IR (KBr) ν_max_ 3218, 2986, 1734, 1636 cm^−1^; ^1^H-NMR (300 MHz, CDCl_3_) *δ* 10.27 (s, 1H), 9.84 (bs, 1H), 8.00 (s, 1H), 7.33 (d, *J* = 8.4 Hz, 1H), 7.13 (dd, *J* = 8.4, 1.5 Hz, 1H), 5.70 (s, 1H), 4.36–4.19 (m, 4H), 2.48 (s, 3H), 1.30 (t, *J* = 7.2 Hz, 6H); ^13^C-NMR (75 MHz, CDCl_3_) *δ* 184.3, 166.4, 136.8, 133.5, 132.7, 126.0, 125.8, 120.0, 114.8, 111.4, 63.0, 49.0, 21.5, 13.9; EIMS *m*/*z* 317 [M]^+^ (100), 271 (62); HRMS (FAB+) *m*/*z* calcd. for C_17_H_20_O_5_N, 318.1341; found: 318.1340.

Diethyl 2-(3-Formyl-5-methoxy-1*H*-indol-2-yl)malonate (**6g**)

Beige solid; yield 68%; mp 112–114 °C; IR (KBr) ν_max_ 3176, 2980, 1732, 1628 cm^−1^; ^1^H-NMR (300 MHz, CDCl_3_) *δ* 10.24 (s, 1H), 9.94 (bs, 1H), 7.69 (d, *J* = 2.4 Hz, 1H), 7.32 (d, *J* = 8.7 Hz, 1H), 6.94 (dd, *J* = 8.9, 2.4 Hz, 1H), 5.64 (s, 1H), 4.36–4.19 (m, 4H), 3.88 (s, 3H), 1.30 (t, *J* = 7.2 Hz, 6H); ^13^C-NMR (75 MHz, CDCl_3_) *δ* 184.1, 166.3, 156.6, 137.1, 130.1, 126.4, 115.2, 114.7, 112.6, 102.0, 63.0, 55.8, 48.9, 13.9; EIMS *m*/*z* 333 [M]^+^ (64), 241 (100); HRMS (FAB+) *m*/*z* calcd. for C_17_H_19_O_6_N, 333.1212; found: 333.1211.

#### 3.1.4. General Procedure for Synthesizing the Methyl 2-(3-Formyl-1*H*-indol-2-yl) Acetate Derivatives (**2a**–**2d** and **2f**–**2g**). This Reaction Procedure Was Designed Based on the Method Described by Somei *et al.* [21], with Slight Changes

To a solution of NaOMe (prepared from sodium, 0.105 g, 4.56 mmol, and anhydrous MeOH, 5 mL) was added a solution containing the corresponding diethyl malonates (**6a**–**6d** and **6f**–**6g**) (0.413 g, 1.30 mmol) in anhydrous MeOH (14 mL). The mixture was then refluxed for 1 h with stirring. After cooling, a saturated solution of NaHCO_3_ was added to the reaction mixture, and the crude product was extracted with CH_2_Cl_2_. The crude extract was washed with anhydrous Na_2_SO_4_ and evaporated under reduced pressure. The residue was purified by column chromatography on a silica gel column using hexane-ethyl acetate as the eluent to obtain the desired product.

Methyl 2-(3-Formyl-1*H*-indol-2-yl)acetate (**2a**)

Brown solid; yield 64%; mp 114–116 °C; IR (KBr) ν_max_ 3187, 2946, 1745, 1629 cm^−1^; ^1^H-NMR (300 MHz, CDCl_3_) *δ* 10.22 (s, 1H), 10.03 (bs, 1H), 8.19–8.14 (m, 1H), 7.42–7.24 (m, 3H), 4.25 (s, 2H), 3.79 (s, 3H); ^13^C-NMR (75 MHz, CDCl_3_) *δ* 184.4, 170.3, 140.2, 135.1, 126.0, 123.7, 122.8, 120.0, 114.6, 111.5, 52.7, 31.3; EIMS *m*/*z* 217 [M]^+^ (23), 185 (100) [22].

Methyl 2-(5-Fluoro-3-formyl-1*H*-indol-2-yl)acetate (**2b**)

Yellow solid; yield 52%; mp 168–170 °C; IR (KBr) ν_max_ 3159, 2952, 1746, 1623 cm^−1^; ^1^H-NMR (300 MHz, CDCl_3_) *δ* 11.78 (bs, 1H), 10.10 (s, 1H), 7.81 (dd, *J* = 9.6, 2.4 Hz, 1H), 7.37 (dd, *J* = 8.7, 4.5 Hz, 1H), 6.97 (dt, *J* = 9.0, 2.4 Hz, 1H), 4.17 (s, 2H), 3.75 (s, 3H); ^13^C-NMR (75 MHz, CDCl_3_) *δ* 183.3, 168.4, 158.6 (d, *J*_CF_ = 235.5 Hz, Ar-quat), 142.2, 131.5, 125.7 (d, *J*_CF_ = 11.3 Hz, Ar-quat), 114.1 (d, *J*_CF_ = 4.5 Hz, Ar-quat), 112.0 (d, *J*_CF_ = 9.8 Hz, Ar-CH), 110.6 (d, *J*_CF_ = 26.3 Hz, Ar-CH), 105.0 (d, *J*_CF_ = 25 Hz, Ar-CH), 51.8, 31.4; EIMS *m*/*z* 235 [M]^+^ (100); HRMS (FAB+) *m*/*z* calcd. for C_12_H_11_O_3_NF, 236.0723; found: 236.0723.

Methyl 2-(5-Chloro-3-formyl-1*H*-indol-2-yl)acetate (**2c**)

Yellow solid; yield 54%; mp 160–162 °C; IR (KBr) ν_max_ 3140, 2945, 1745, 1637 cm^−1^; ^1^H-NMR (300 MHz, CDCl_3_ + DMSO-*d*_6_) *δ* 11.60 (bs, 1H), 10.12 (s, 1H), 8.16 (d, *J* = 2.1 Hz, 1H), 7.35 (d, *J* = 8.7 Hz, 1H), 7.18 (dd, *J* = 8.7, 2.1 Hz, 1H), 4.16 (s, 2H), 3.75 (s, 3H); ^13^C-NMR (75 MHz, CDCl_3_ + DMSO-*d*_6_) *δ* 183.7, 168.9, 142.1, 133.8, 127.8, 126.6, 123.3, 119.6, 114.0, 112.5, 52.2, 31.7; EIMS *m*/*z* 251 [M]^+^ (85), 252 (20), 253 (34), 254 (7); HRMS (FAB+) *m*/*z* calcd. for C_12_H_11_O_3_NCl, 252.0427; found: 252.0422.

Methyl 2-(5-Bromo-3-formyl-1*H*-indol-2-yl)acetate (**2d**)

Yellow solid; yield 32%; mp 172–174 °C; IR (KBr) ν_max_ 3132, 2945, 1745, 1637 cm^−1^; ^1^H-NMR (300 MHz, CDCl_3_ + DMSO-*d*_6_) *δ* 11.06 (bs, 1H), 10.15 (s, 1H), 8.33 (d, *J* = 1.8 Hz, 1H), 7.34 (dd, *J* = 8.5, 1.8 Hz, 1H), 7.28 (dd, *J* = 8.7, 0.6 Hz, 1H), 4.19 (s, 2H), 3.77 (s, 3H); ^13^C-NMR (75 MHz, CDCl_3_ + DMSO-*d*_6_) *δ* 183.9, 169.4, 141.6, 134.1, 127.5, 126.3, 122.9, 116.0, 114.1, 113.0, 52.6, 31.7; EIMS *m*/*z* 295 [M]^+^ (76), 297 (72); HRMS (FAB+) *m*/*z* calcd. for C_12_H_11_O_3_NBr, 295.9922; found: 295.9926.

Methyl 2-(3-Formyl-5-methyl-1*H*-indol-2-yl)acetate (**2f**)

Yellow solid; yield 23%; mp 124–126 °C; IR (KBr) ν_max_ 3164, 2920, 1738, 1617 cm^−1^; ^1^H-NMR (300 MHz, CDCl_3_) *δ* 10.20 (s, 1H), 9.85 (bs, 1H), 7.98 (s, 1H), 7.28 (d, *J* = 8.1 Hz, 1H), 7.09 (d, *J* = 8.4 Hz, 1H), 4.24 (s, 2H), 3.80 (s, 3H), 2.46 (s, 3H); ^13^C-NMR (75 MHz, CDCl_3_) *δ* 184.3, 170.4, 140.1, 133.4, 132.5, 126.2, 125.2, 119.9, 114.3, 111.1, 52.7, 31.3, 21.5; EIMS *m*/*z* 231 [M] ^+^ (100), 199 (85); HRMS (FAB+) *m*/*z* calcd. for C_13_H_13_O_3_N, 231.0895; found: 231.0898.

Methyl 2-(3-Formyl-5-methoxy-1*H*-indol-2-yl)acetate (**2g**)

Beige solid; yield 64%; mp 126–128 °C; IR (KBr) ν_max_ 3314, 2950, 1727, 1641 cm^−1^; ^1^H-NMR (300 MHz, CDCl_3_) *δ* 10.16 (s, 1H), 9.92 (bs, 1H), 7.67 (d, *J* = 2.4 Hz, 1H), 7.27 (d, *J* = 9.0 Hz, 1H), 6.89 (dd, *J* = 8.8, 2.7 Hz, 1H), 4.21 (s, 2H), 3.87 (s, 3H), 3.79 (s, 3H); ^13^C-NMR (75 MHz, CDCl_3_) *δ* 184.1, 170.2, 156.5, 140.5, 129.9, 126.7, 114.7, 113.9, 112.2, 102.1, 55.7, 52.7, 31.2; EIMS *m*/*z* 247 [M]^+^ (82), 215 (100); HRMS (FAB+) *m*/*z* calcd. for C_13_H_13_O_4_N, 247.0845; found: 247.0836.

#### 3.1.5. General Procedure for Synthesizing **2d** and **2e**

To a solution of the diethyl indole malonate (**6d** or **6e**) (0.350 g, 0.815 mmol) in ethanol (2.3 mL) was added a solution of KOH (2 M) (1.8 mL, 3.67 mmol). The solution was then stirred at room temperature for 21 h. A solution of HCl 2 N was then added and refluxed for 2 h. Subsequently, 20 mL of a saturated solution of Na_2_CO_3_ were added to the reaction mixture. The crude product was extracted with ethyl acetate, and the organic phases were combined and concentrated under reduced pressure. The esterification step was accomplished by hydrolyzing and decarboxylating the residue. This transformation was accomplished by dissolving crude acetic acid (0.213 g, 0.647 mmol) in DMF (1.5 mL). Potassium bicarbonate (KHCO_3_) (0.097 g, 0.970 mmol) and MeI (0.137 g, 0.970 mmol) were added, and the reaction was stirred for 5 h at room temperature. To the reaction mixture was added 20 mL of a solution containing 1 M potassium bisulfate (KHSO_4_), followed by the addition of ethyl acetate. The crude product was then extracted. The organic phases were combined and washed with an aqueous saturated solution of sodium bicarbonate (NaHCO_3_) and brine. The organic extract was dried over Na_2_SO_4_ and concentrated under reduced pressure, and the crude product was purified by column chromatography using silica gel and a mixture hexane–ethyl acetate as the eluent to obtain the desired product [22,23].

Methyl 2-(5-Bromo-3-formyl-1*H*-indol-2-yl)acetate (**2d**)

Yellow solid; yield 48%; mp 172–174 °C.

Methyl 2-(3-Formyl-5-iodo-1*H*-indol-2-yl)acetate (**2e**)

Yellow solid; yield 60%; mp 168–170 °C; IR (KBr) ν_max_ 3129, 2947, 2922, 1747, 1637 cm^−1^; ^1^H-NMR (300 MHz, CDCl_3_ + DMSO-*d*_6_) *δ* 11.73 (bs, 1H), 10.11 (s, 1H), 8.53 (s, 1H), 7.49 (dd, *J* = 8.5, 1.5 Hz, 1H), 7.22 (d, *J* = 8.4 Hz, 1H), 4.16 (s, 2H), 3.75 (s, 3H); ^13^C-NMR (75 MHz, CDCl_3_+ DMSO-*d*_6_) *δ* 183.6, 168.6, 141.6, 134.4, 131.2, 128.7, 127.6, 113.4, 113.3, 86.0, 52.1, 31.5; EIMS *m*/*z* 343 [M]^+^ (100), 311 (93); HRMS (FAB+) *m*/*z* calcd. for C_12_H_11_O_3_NI, 343.9784; found: 343.9786.

#### 3.1.6. General Procedure for Synthesizing Caulerpin **1a** and Its Analogues (**1b**–**1g**)

To a solution of the corresponding indole (**2a**–**2g**) (0.100 g, 0.432 mmol) in anhydrous xylene (14 mL/0.1mmol) were added piperidine (0.128 mL, 1.30 mmol) and diethylamine (0.134 mL, 1.30 mmol), and the mixture was refluxed during the removal of water using a Dean-Stark separator. After the starting material had been consumed, as indicated by TLC analysis, the solvent was concentrated in vacuum. The residue was purified by column chromatography using silica gel and a mixture of hexane-ethyl acetate as the eluent to obtain the desired product.

Dimethyl 5,12-Dihydrocycloocta[1,2-*b*:5,6-*b′*]-diindole-6,13-dicarboxylate (**1a**)

Red solid; yield 32%; mp 318 °C; IR (KBr) ν_max_ 3377, 2917, 1681 cm^−1^; ^1^H-NMR (300 MHz, CDCl_3_ + DMSO-*d*_6_) *δ* 10.74 (bs, 2H), 8.18 (s, 2H), 7.42–7.32 (m, 4H), 7.12–7.03 (m, 4H), 3.84 (s, 6H); ^13^C-NMR (75 MHz, CDCl_3_ + DMSO-*d*_6_) *δ* 165.8, 141.7, 137.3, 132.5, 127.0, 125.3, 122.2, 119.6, 117.2, 111.3, 111.1, 51.7; EIMS *m*/*z* 398 [M]^+^ (100); HRMS (FAB+) *m*/*z* calcd. for C_24_H_18_O_4_N_2_, 398.1267; found: 398.1266.

Dimethyl 2,9-Difluoro-5,12-dihydrocycloocta[1,2-*b*:5,6-*b′*]-diindole-6,13-dicarboxylate (**1b**)

Red solid; yield 24%; mp 340–342 °C; IR (KBr) ν_max_ 3373, 2952, 2918, 1678 cm^−1^; ^1^H-NMR (300 MHz, CDCl_3_ + DMSO-*d*_6_) *δ* 11.16 (bs, 2H), 8.09 (s, 2H), 7.32 (dd, *J* = 8.7, 4.5 Hz, 2H), 7.07 (dd, *J* = 9.3, 2.4 Hz, 2H), 6.90 (dt, *J* = 9.0, 2.4 Hz, 2H), 3.83 (s, 6H); ^13^C-NMR (75 MHz, CDCl_3_ + DMSO-*d*_6_) *δ* 164.5, 158.0, 154.9, 140.0, 133.0 (d, *J*_CF_ = 15 Hz, Ar-quat), 126.0 (d, *J*_CF_ = 9.8 Hz, Ar-quat), 124.3, 111.5 (d, *J*_CF_ = 9 Hz, Ar-CH), 109.9 (d, *J*_CF_ = 4.5 Hz, Ar-quat), 109.4 (d, *J*_CF_ = 26 Hz, Ar-CH), 101.3 (d, *J*_CF_ = 23.3 Hz, Ar-CH), 50.8; EIMS *m*/*z* 434 [M]^+^ (100); HRMS (FAB+) *m*/*z* calcd. for C_24_H_16_O_4_N_2_F_2_, 434.1078; found: 434.1081.

Dimethyl 2,9-Chloro-5,12-dihydrocycloocta[1,2-*b*:5,6-*b′*]-diindole-6,13-dicarboxylate (**1c**)

Red solid; yield 17%; mp 350–352 °C; IR (KBr) ν_max_ 3378, 2955, 2922, 1686 cm^−1^; ^1^H-NMR (300 MHz, CDCl_3_ + DMSO-*d*_6_) *δ* 10.67 (bs, 2H), 8.09 (s, 2H), 7.38 (d, *J* = 2.1 Hz, 2H), 7.29 (d, *J* = 8.4 Hz, 2H), 7.08 (dd, *J* = 8.4, 2.1 Hz, 2H), 3.86 (s, 6H); ^13^C-NMR (75 MHz, CDCl_3_ + DMSO-*d*_6_) *δ* 165.6, 141.2, 135.7, 133.6, 127.9, 125.4, 125.2, 122.4, 116.7, 112.5, 110.7, 51.8; EIMS *m*/*z* 466 [M]^+^ (100), 467 (30), 468 (67), 469 (20), 470 (14); HRMS (FAB+) *m*/*z* calcd. for C_24_H_16_O_4_N_2_Cl_2_, 466.0487; found: 466.0484.

Dimethyl 2,9-Bromo-5,12-dihydrocycloocta[1,2-*b*:5,6-*b′*]-diindole-6,13-dicarboxylate (**1d**)

Red solid; yield 32%; mp 340–342 °C; IR (KBr) ν_max_ 3374, 2919, 2850, 1679 cm^−1^; ^1^H-NMR (300 MHz, CDCl_3_ + DMSO-*d*_6_) *δ* 11.01 (bs, 2H), 8.10 (s, 2H), 7.53 (d, *J* = 1.5 Hz, 2H), 7.26 (d, *J* = 8.4 Hz, 2H), 7.19 (dd, *J* = 8.7, 1.8 Hz, 2H), 3.85 (s, 6H); ^13^C-NMR (75 MHz, CDCl_3_ + DMSO-*d*_6_) *δ* 164.9, 140.5, 135.5, 133.0, 127.9, 125.1, 124.4, 119.3, 112.6, 112.2, 110.0, 51.3; EIMS *m*/*z* 554 [M]^+^ (54), 556 (100), 558 (53); HRMS (FAB+) *m*/*z* calcd. for C_24_H_16_O_4_N_2_^79^Br^81^Br, 555.9456; found: 555.9466.

Dimethyl 2,9-Iodo-5,12-dihydrocycloocta[1,2-*b*:5,6-*b′*]-diindole-6,13-dicarboxylate (**1e**)

Red solid; yield 10%; mp 318–320 °C; IR (KBr) ν_max_ 3377, 2917, 2850, 1677 cm^−1^; ^1^H-NMR (300 MHz, CDCl_3_ + DMSO-*d*_6_) *δ* 11.03 (bs, 2H), 8.09 (s, 2H), 7.72 (s, 2H), 7.36 (dd, *J* = 8.4, 1.5 Hz, 2H), 7.16 (d, *J* = 8.7 Hz, 2H), 3.84 (s, 6H); ^13^C-NMR (75 MHz, CDCl_3_ + DMSO-*d*_6_) *δ* 164.8, 140.4, 135.8, 132.6, 129.7, 128.7, 125.5, 125.0, 113.0, 109.6, 82.5, 51.2; EIMS *m*/*z* 650 [M]^+^ (28), 649 (100); HRMS (FAB+) *m*/*z* calcd. for C_24_H_16_O_4_N_2_I_2_, 649.9200; found: 649.9208.

Dimethyl 2,9-Dimethyl-5,12-dihydrocycloocta[1,2-*b*:5,6-*b′*]-diindole-6,13-dicarboxylate (**1f**)

Red solid; yield 24%; mp 288–290 °C; IR (KBr) ν_max_ 3382, 2913, 2852, 1680 cm^−1^; ^1^H-NMR (300 MHz, CDCl_3_ + DMSO-*d*_6_) *δ* 10.56 (bs, 2H), 8.13 (s, 2H), 7.22 (d, *J* = 8.1 Hz, 2H), 7.18 (s, 2H), 6.94 (dd, *J* = 8.3, 1.5 Hz, 2H), 3.83 (s, 6H), 2.39 (s, 6H); ^13^C-NMR (75 MHz, CDCl_3_ + DMSO-*d*_6_) *δ* 165.3, 141.1, 135.1, 131.9, 128.2, 126.7, 124.5, 123.3, 116.4, 110.5, 110.2, 51.1, 20.4; EIMS *m*/*z* 426 [M]^+^ (100); HRMS (FAB+) *m*/*z* calcd. for C_26_H_22_O_4_N_2_, 426.1580; found: 426.1573.

Dimethyl 2,9-Dimethoxy-5,12-dihydrocycloocta[1,2-*b*:5,6-*b′*]-diindole-6,13-dicarboxylate (**1g**)

Red solid; yield 23%; mp 306–308 °C; IR (KBr) ν_max_ 3343, 2947, 2924, 1695 cm^−1^; ^1^H-NMR (300 MHz, CDCl_3_ + DMSO-*d*_6_) *δ* 10.43 (bs, 2H), 8.11 (s, 2H), 7.24 (d, *J* = 8.7 Hz, 2H), 6.83 (d, *J* = 2.4 Hz, 2H), 6.77 (dd, *J* = 8.7, 2.4 Hz, 2H), 3.85 (s, 6H), 3.82 (s, 6H); ^13^C-NMR (75 MHz, CDCl_3_ + DMSO-*d*_6_) *δ* 165.7, 153.8, 141.3, 132.6, 132.1, 127.2, 124.5, 112.3, 111.9, 110.6, 98.8, 54.9, 51.4; EIMS *m*/*z* 458 [M]^+^ (100); HRMS (FAB+) *m*/*z* calcd. for C_26_H_22_O_6_N_2_, 458.1478; found: 458.1479.

### 3.2. Anti-Tuberculosis Activity

Stock and working solutions. Stock solutions of all compounds were prepared in 100% dimethyl sulfoxide (DMSO) at a concentration of 20,000 µM and were sterilized by filtration through a 0.22 µm PTFE membrane. The susceptibility tests were conducted using Microplate alamar Blue by preparing the working solutions from stock solutions diluted in sterile 7H9 broth to a final concentration of 100 µM.

Antimycobacterial activity. Antimicrobial susceptibility tests were performed in 96-well microplates according to the methods described by Collins and Franzblau (1998) [29]. Outer perimeter wells were filled with sterile distilled water (200 µL) to prevent the dehydration of the experimental wells. Colum 2 (B to G wells) was used to evaluate the reference drug rifampin. Serial two-fold dilutions were prepared in 100 µL of the Middlebrook 7H9 medium (2.0 to 0.006 µg/mL) in the microplates. Wells 10E and 10F were used as DMSO controls. Four wells (11B–11E) were used as drug-free controls and received 100 µL of the supplemented 7H9 broth and 100 µL of the bacterial inoculum (1 × 10^6^ cfu/mL). A 1:100 diluted control was prepared from the bacterial suspension to test the growth of a 1% bacterial solution.

All other wells received 100 µL of the working solution (100 µM) and 100 µL bacterial inoculum. The final concentration of DMSO in each well was 1.0% v/v. All compounds were first tested at 50 µM, and the IC_50_ values were calculated only for extracts that inhibited ≥75% of the mycobacterial growth. These compounds were tested at different concentrations (**1a**: 50–0.097 µM; **1b**: 50–0.78 µM; **1d**: 25–0.78 µM; **1e**: 50–3.125 µM) in increments of 0.3 logs. Each microplate was incubated for 7 days at 37 °C. After incubation, one control sample was diluted into a mixture of 20 µL alamar Blue (ABD Serotec) and 5 µL of a sterile 20% Tween 80 solution. The plates were re-incubated at 37 °C for 24 h. After this incubation period, any wells that changed from blue to pink received a mixture of alamar-Tween and were incubation for an additional 24 h.

The percent reduction of alamar Blue was calculated according to the manufacturer’s protocol. The optical densities of the microplate wells were measured at 540 and 600 nm using a spectrophotometer. The percent inhibition of the compounds was defined as the concentration that yielded complete reduction of the alamar Blue.

## 4. Conclusions

We successfully developed a synthetic approach for the preparation of **1a** in three steps with an overall yield of 11%. Six new analogues of **1a** were synthesized from the indoles in four steps with overall yields between 3% and 8%. Although these yields are relatively low, this is the first report of the synthesis of a series of bis-indole analogues of **1a** in a few reaction steps. Our biological results indicated that **1a** may be useful as a lead compound for the development of novel anti-tuberculosis agents. Unfortunately, the compounds **1b**–**1g** did not display appreciable anti-tuberculosis activities. Therefore, future studies will focus on determining the mechanism by which these new compounds inhibit anti-tuberculosis growth. We will additionally test the effects of combinations of appropriately positioned substituents.

## Supplementary Files

Supplementary File 1 Supplementary Information (PDF, 814 KB)
