# Supplementary material for: Synthesis and Anti-Tuberculosis Activity of the Marine Natural Product Caulerpin and Its Analogues
Source: Mar Drugs. 2014 Mar 27;12(4):1757–72. doi: 10.3390/md12041757 (PMC4012461; doi:10.3390/md12041757)
Supplement: Supplementary File 1 — Supplementary Information (PDF, 814 KB) [file marinedrugs-12-01757-s001.pdf]

Supplementary Information

Scheme S1. Optimization of the reaction conditions for the synthesis of **1a**.

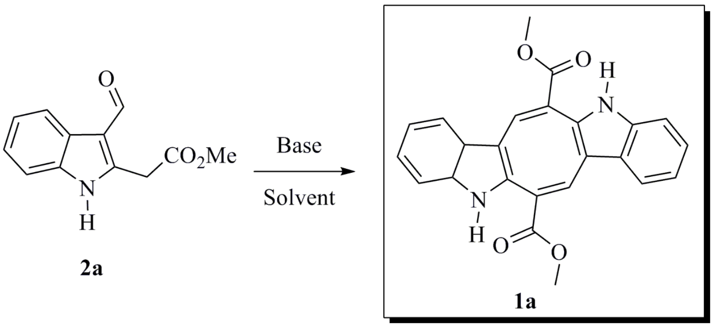

Table S1. Optimization of the reaction conditions for the synthesis of **1a**.

Entry	Base	Concentration (M)	Solvent	Temperature (°C)	Time (h)	Yield (%)
1	Diethylamine Piperidine	0.01	Methanol	reflux	48	2[9]
2	Diethylamine Piperidine	0.01	Xylene	reflux	3	12
3	Diethylamine Piperidine	0.023	Xylene	reflux	3	32
4	Diethylamine Piperidine	0.038	Xylene	reflux	3	31
5	Diethylamine Piperidine	0.076	Xylene	reflux	4	32
6	Diethylamine Piperidine	0.230	Xylene	reflux	6	15
7	Diethylamine Piperidine	0.076	Toluene	reflux	4.5	24
8	Diethylamine Piperidine	0.230	Toluene	reflux	7.5	12

Anti-Tuberculosis Activity

Figure S1. IC_{50} values of compounds **1a**, **1b**, **1d** and **1e** against the *M. tuberculosis* strain H37Rv. Values are the means ± S.E.M., *n* = 5.

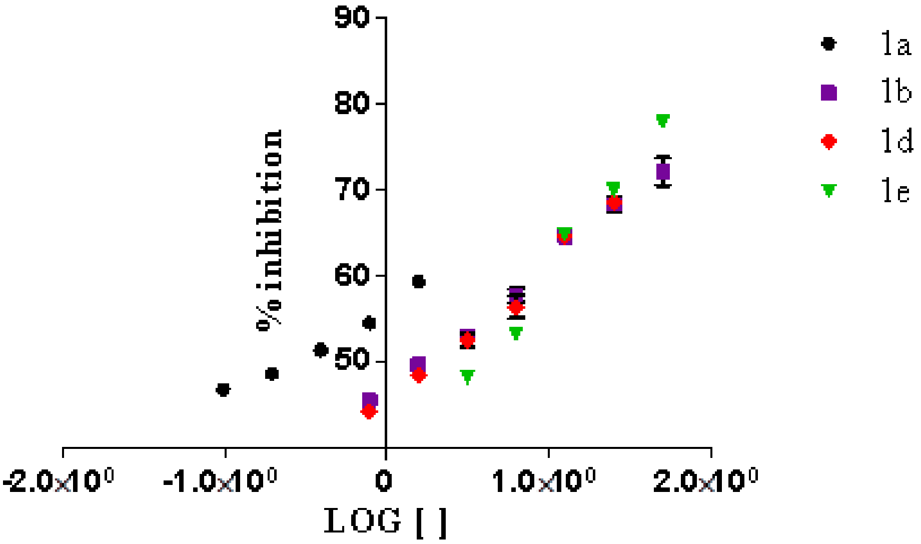

Figure S2. Concentration-response curve of compound **1a** against the *M. tuberculosis* strain H37Rv. This compound was tested at a concentration of 50–1.56 μM. Values are the means ± S.E.M., *n* = 5.

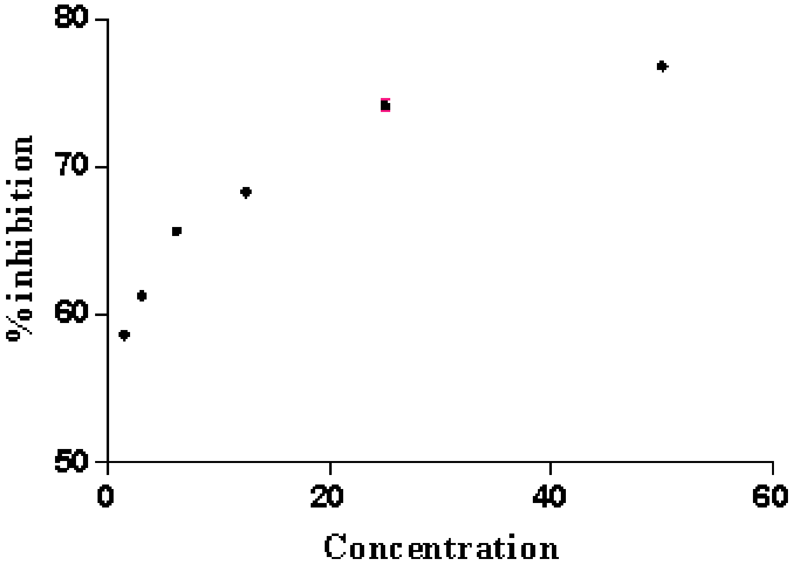

Figure S3. IC_{50} values of rifampin against the *M. tuberculosis* strain H37Rv. Values are the means ± S.E.M., n = 5.

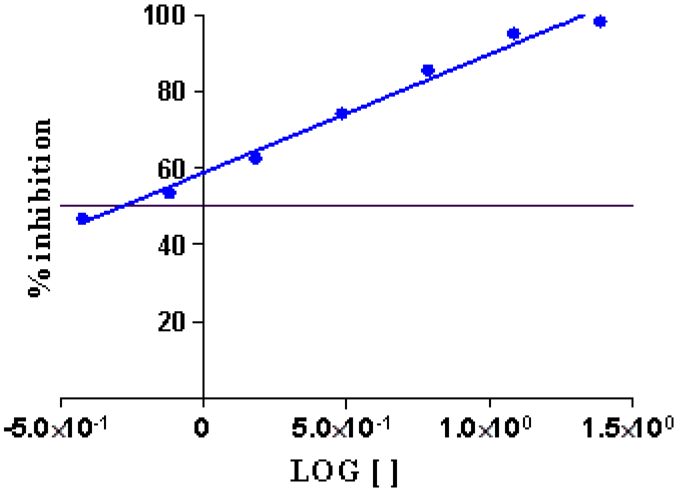

Figure S4. ^{1}H NMR spectrum of the Caulerpin (**1a**).

Caulerpin 1H NMR (300 MHz, CDCl3 + DMSO-d6)

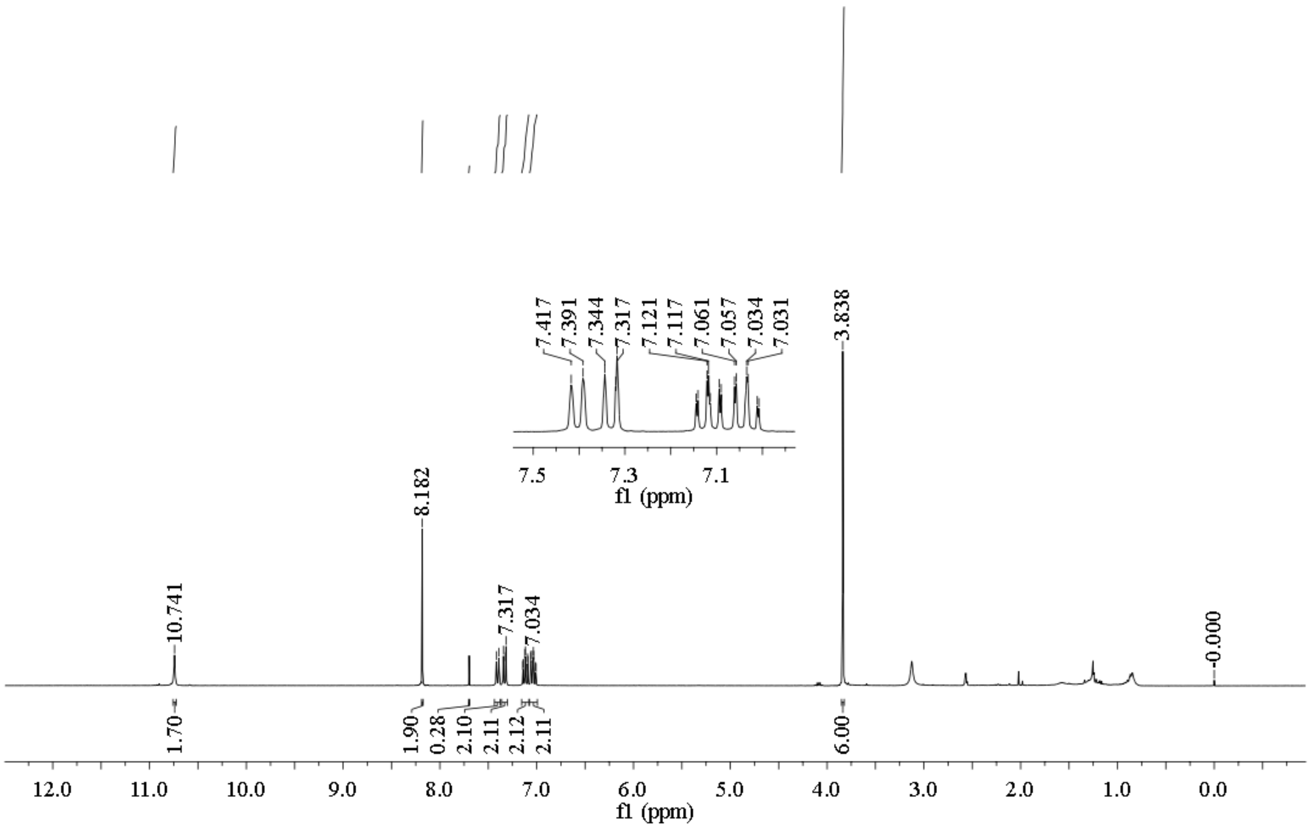

Figure S5. ^{13}C NMR spectrum of the Caulerpin (**1a**).

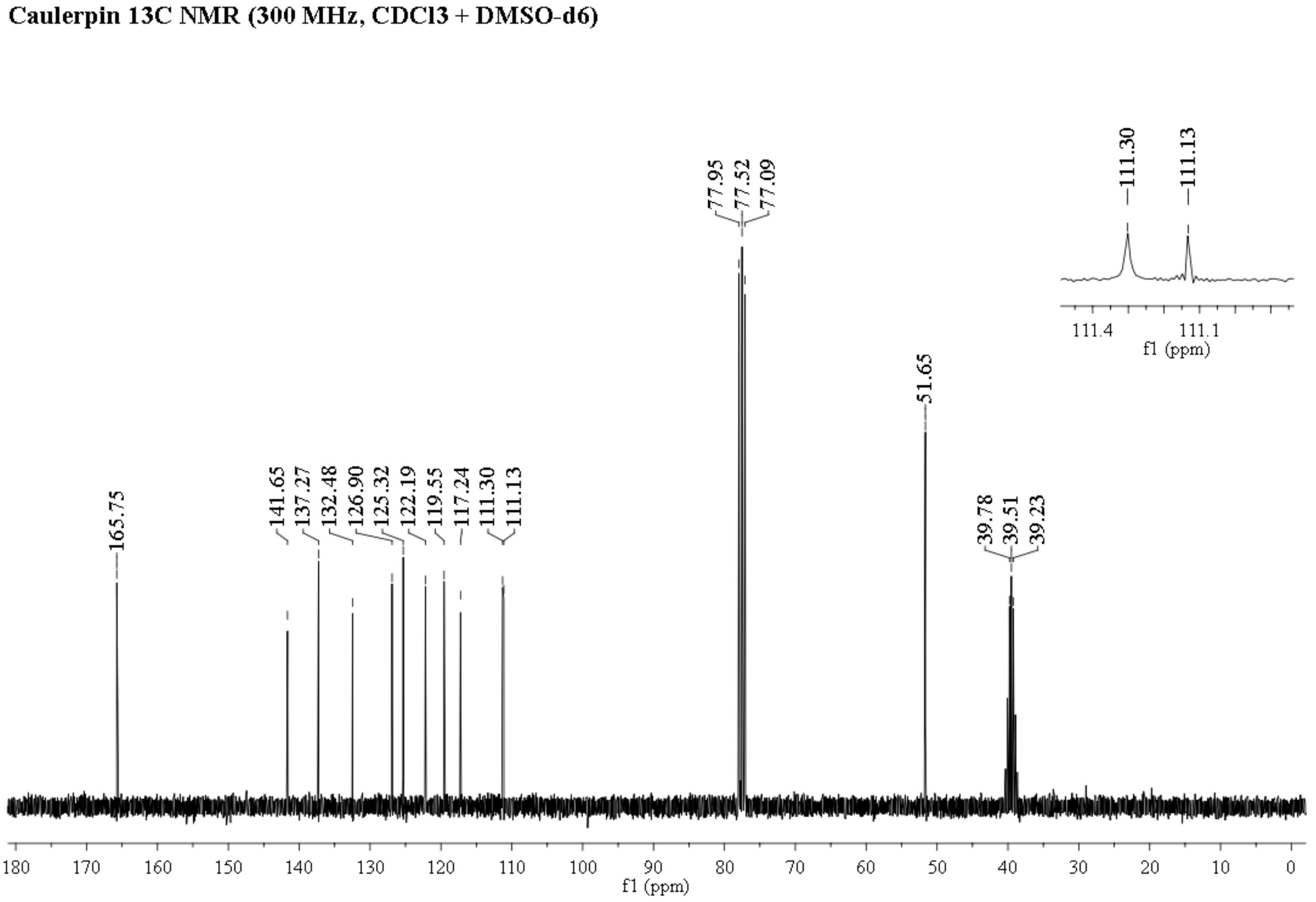

Figure S6. Elemental composition of the Caulerpin (**1a**).

```
[ Elemental Composition ]                                        Page: 1
Data   : Dr-Roberto-Martinez003        Date : 02-Dec-2013 13:24
Sample: 2616   1a
Note : -luis-velasco
Inlet : Direct                         Ion Mode : FAB+
RT : 1.04 min                          Scan#: (2,7)
Elements : C 40/0, H 49/0, O 5/0, N 3/1
Mass Tolerance      : 1000ppm, 1mmu if m/z > 1
Unsaturation (U.S.) : -0.5 - 20.0

Observed m/z   Int%
  398.1266     100.0
Estimated m/z  Error[ppm]  U.S.    C    H    O    N
  398.1267       -0.3      17.0   24   18    4    2
```

Figure S7. Mass spectrum of the Caulerpin (**1a**).

Caulerpin, 1a

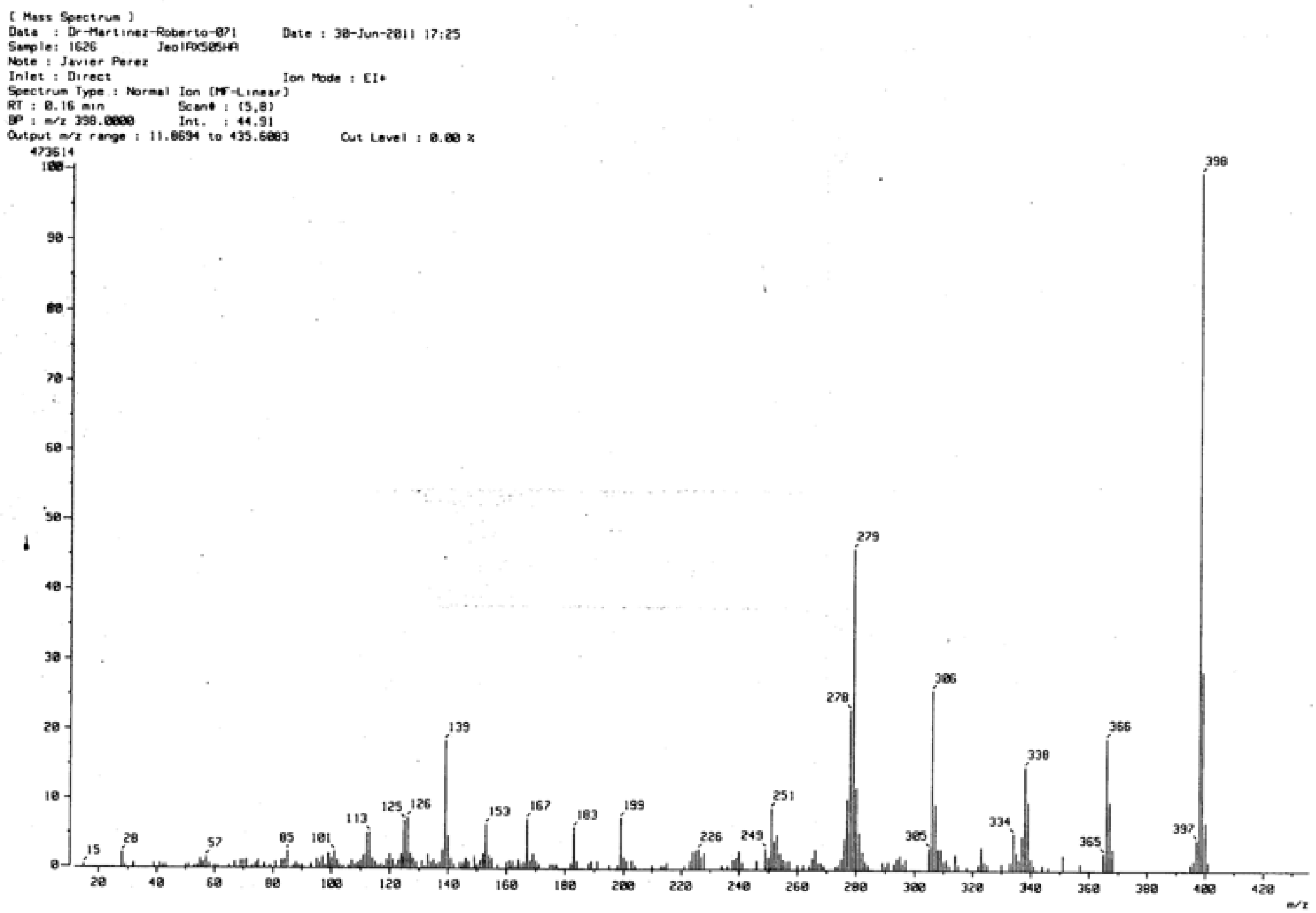

Figure S8. ^{1}H NMR spectrum of diethyl 2-(3-formyl-1*H*-indol-2-yl)malonate (**6a**).

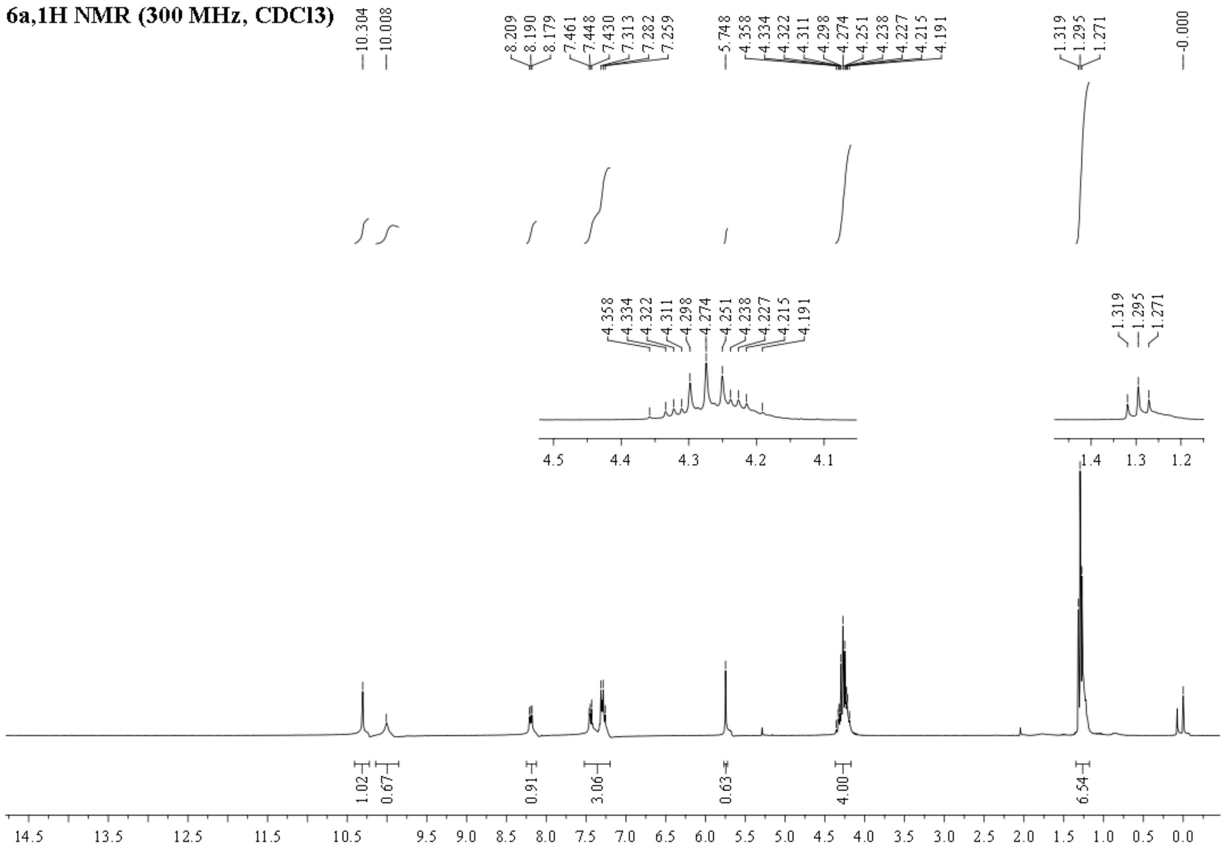

Figure S9. ^{13}C NMR spectrum of diethyl 2-(3-formyl-1*H*-indol-2-yl)malonate (**6a**).

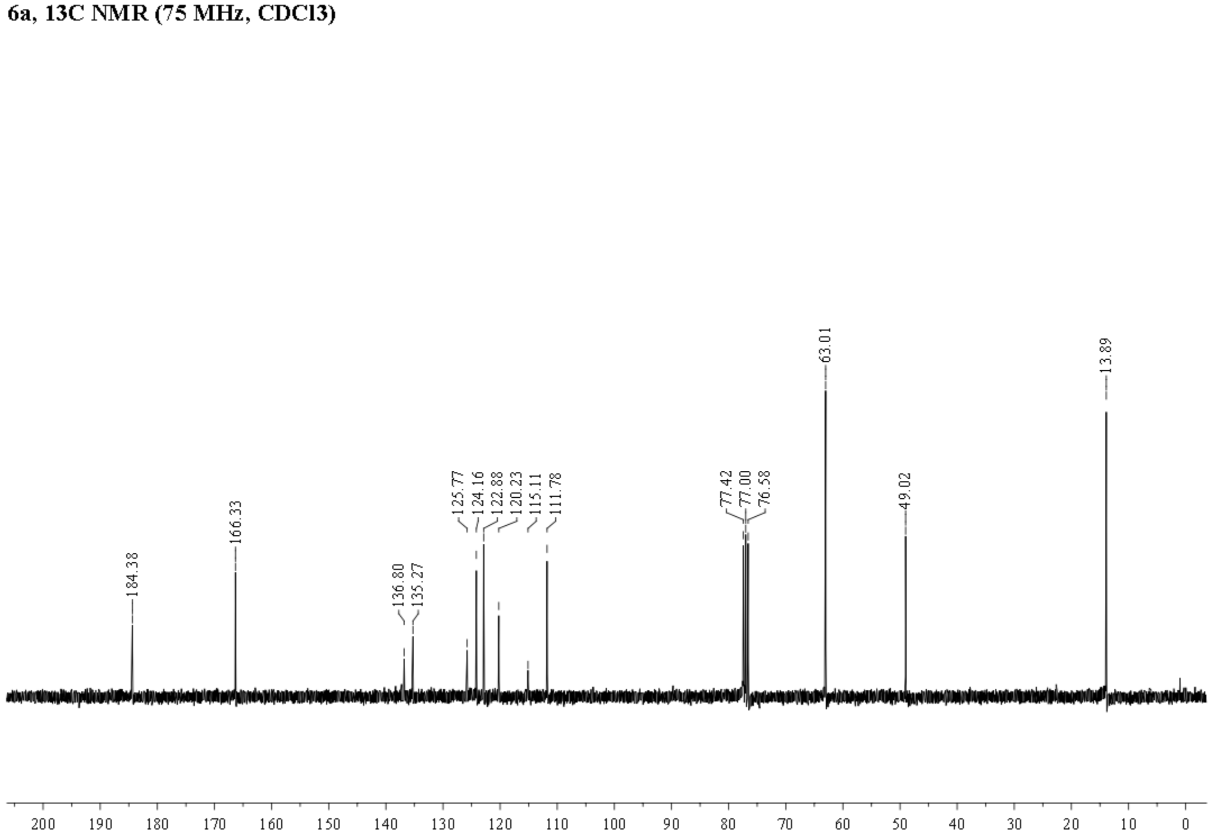

Figure S10. Mass spectrum of diethyl 2-(3-formyl-1*H*-indol-2-yl)malonate (**6a**).

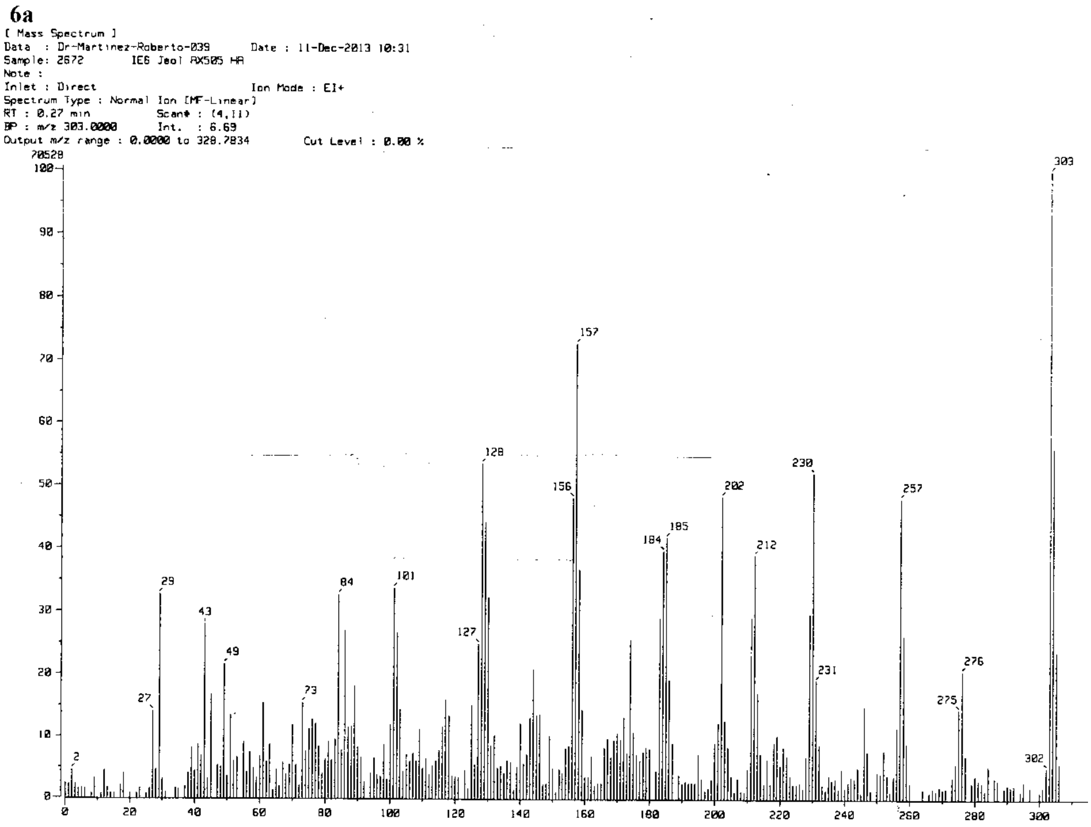

Figure S11. ^{1}H NMR spectrum of dimethyl 2,9-dimethyl-5,12-dihydrocycloocta [1,2-*b*:5,6-*b′*]-diindole-6,13-dicarboxylate (**1f**).

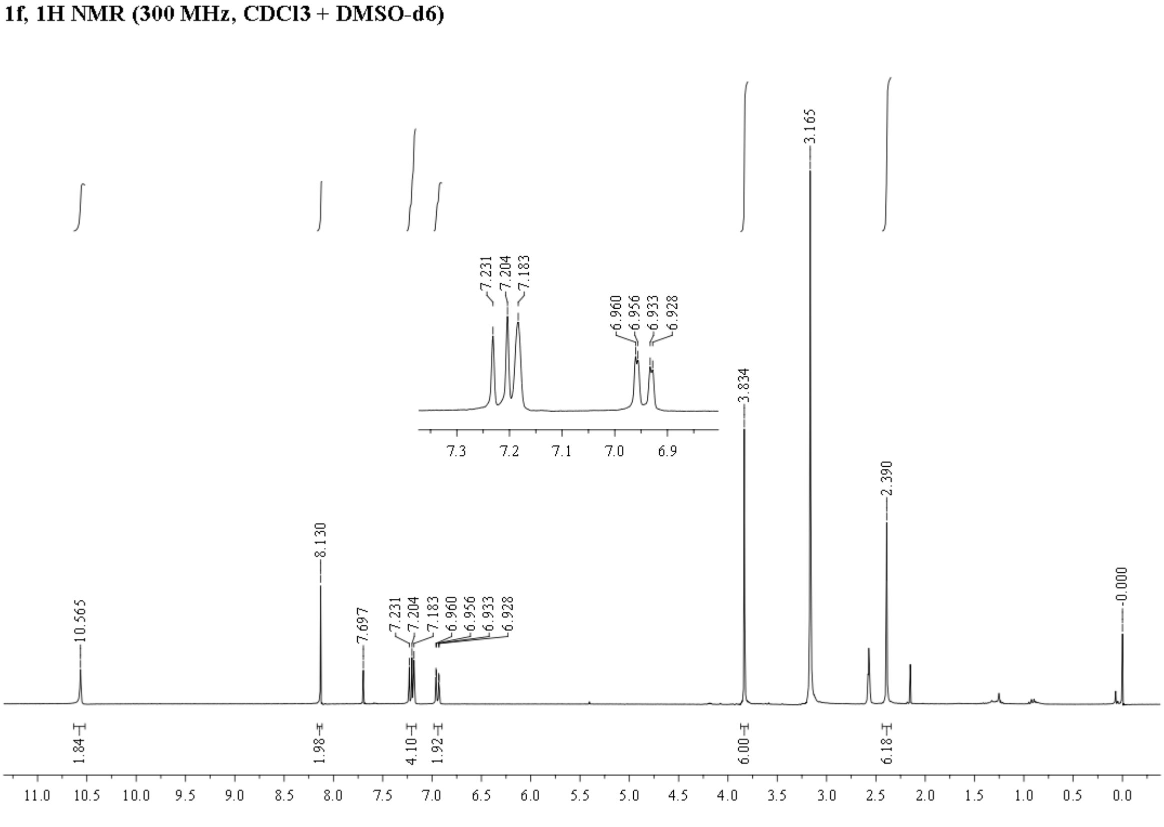

Figure S12. ^{13}C NMR spectrum of dimethyl 2,9-dimethyl-5,12-dihydrocycloocta [1,2-*b*:5,6-*b′*]-diindole-6,13-dicarboxylate (**1f**).

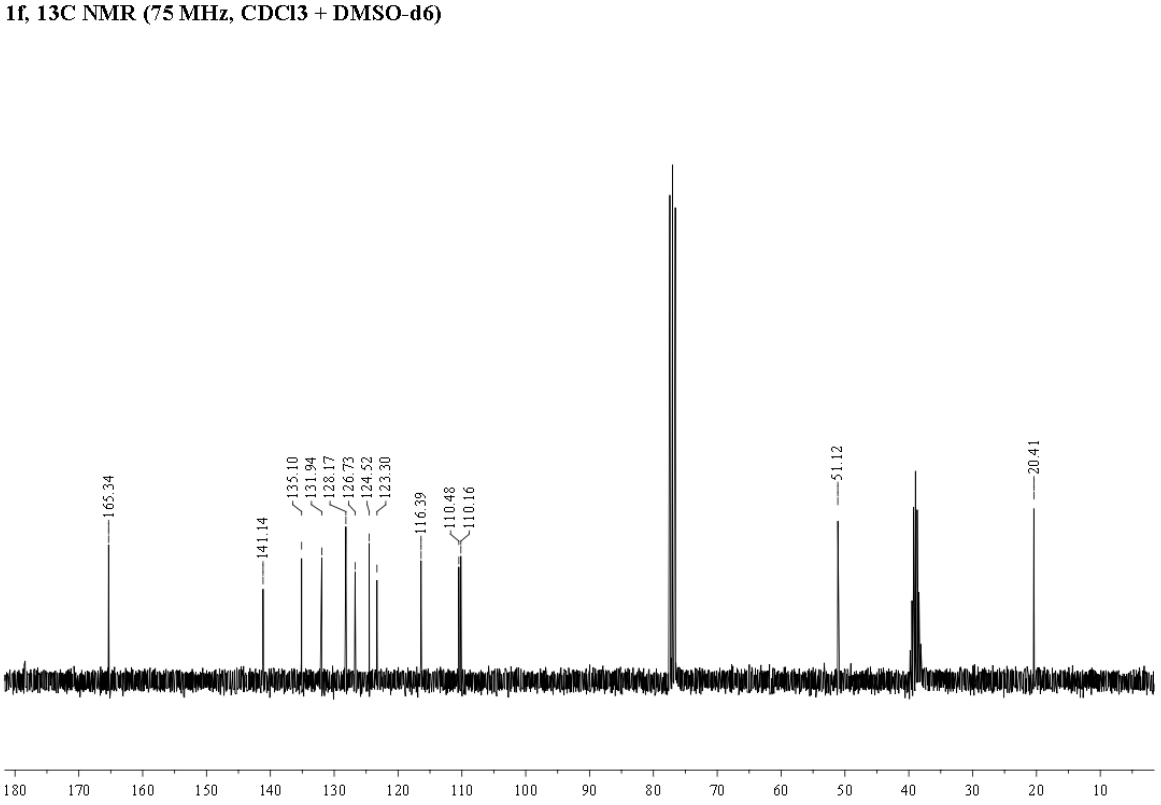

Figure S13. Mass spectrum of dimethyl 2,9-dimethyl-5,12-dihydrocycloocta [1,2-*b*:5,6-*b′*]-diindole-6,13-dicarboxylate (**1f**).

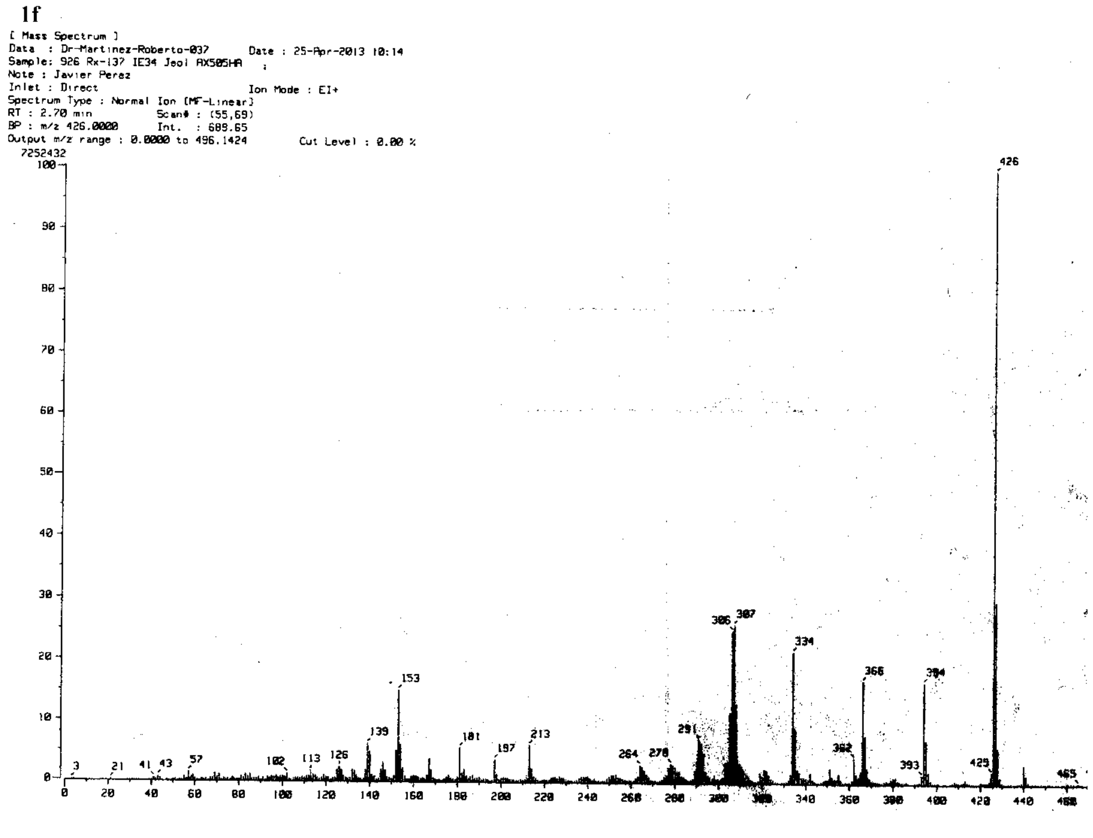

Figure S14. Elemental composition of dimethyl 2,9-dimethyl-5,12-dihydrocycloocta [1,2-*b*:5,6-*b'*]-diindole-6,13-dicarboxylate (**1f**).

1f

```
[ Elemental Composition ]                                        Page: 1
Data   : Dr-Roberto-Martinez026       Date : 11-Jun-2013 14:00
Sample: 1351     Rx-137
Note : -luis-velasco
Inlet : Direct                         Ion Mode : FAB+
RT : 2.67 min                          Scan#: (9,13)+(4,6)
Elements : C 40/0, H 49/0, O 7/0, N 3/0
Mass Tolerance      : 1000ppm, 1mmu if m/z > 1
Unsaturation (U.S.) : 0.0 - 34.0

Observed m/z    Int%
  426.1573      100.0
Estimated m/z   Error[ppm]   U.S.     C     H     O     N
  426.1580        -1.6       17.0    26    22     4     2
```

Figure S15. ^{1}H NMR spectrum of diethyl 2-(3-formyl-5-methyl-1*H*-indol-2-yl)malonate (**6f**).

6f, 1H NMR (300 MHz, CDCl3)

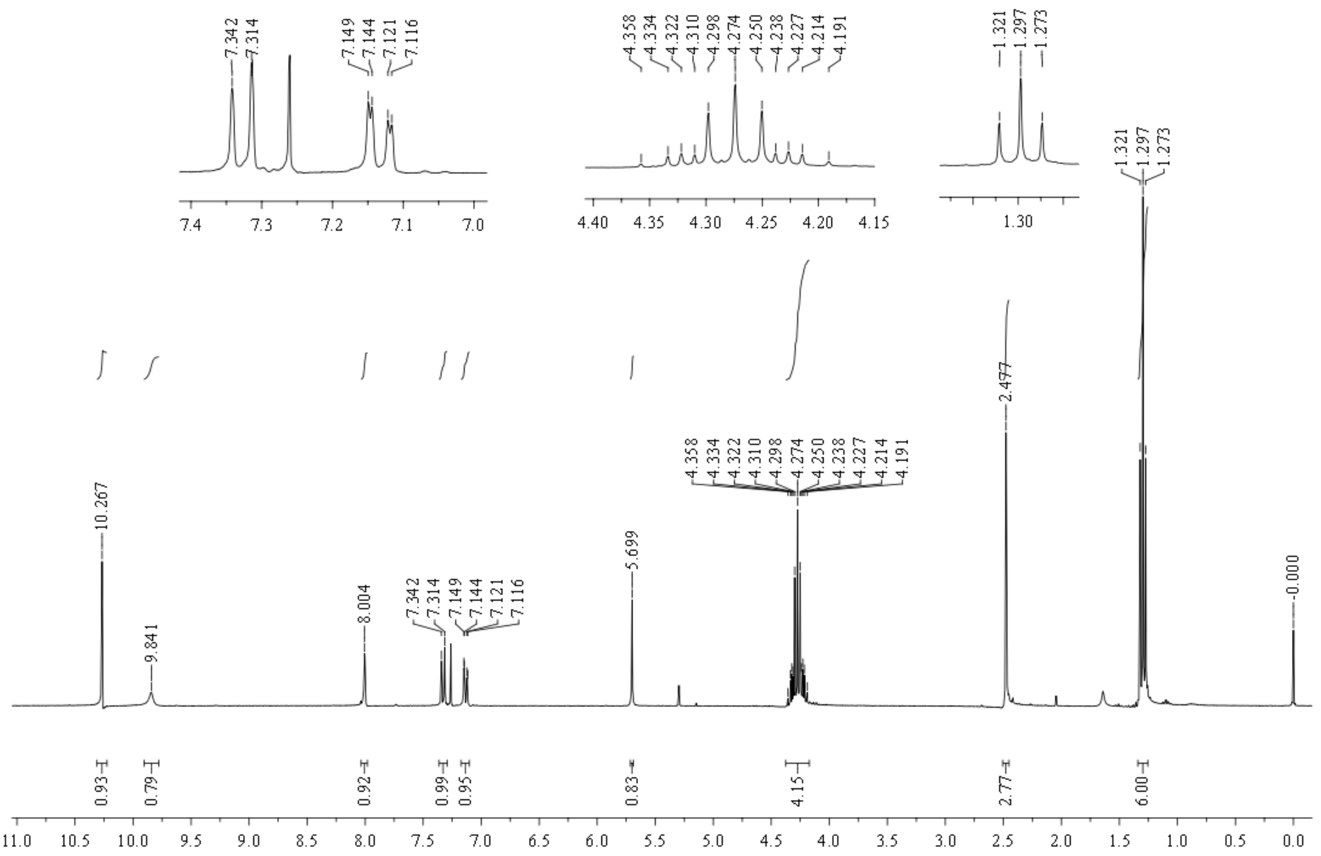

Figure S16. ^{13}C NMR spectrum of diethyl 2-(3-formyl-5-methyl-1*H*-indol-2-yl)malonate (**6f**).

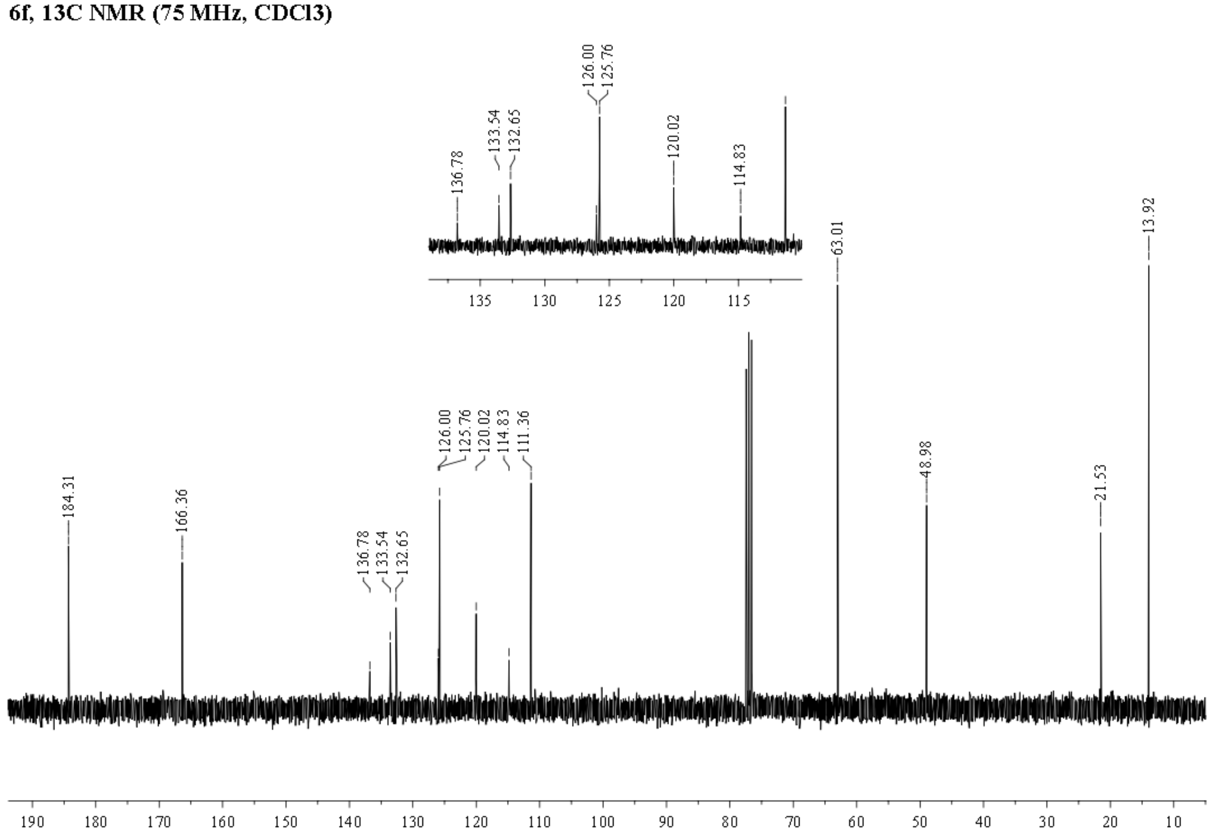

Figure S17. ^{1}H NMR spectrum of methyl 2-(3-formyl-5-methyl-1*H*-indol-2-yl)acetate (**2f**).

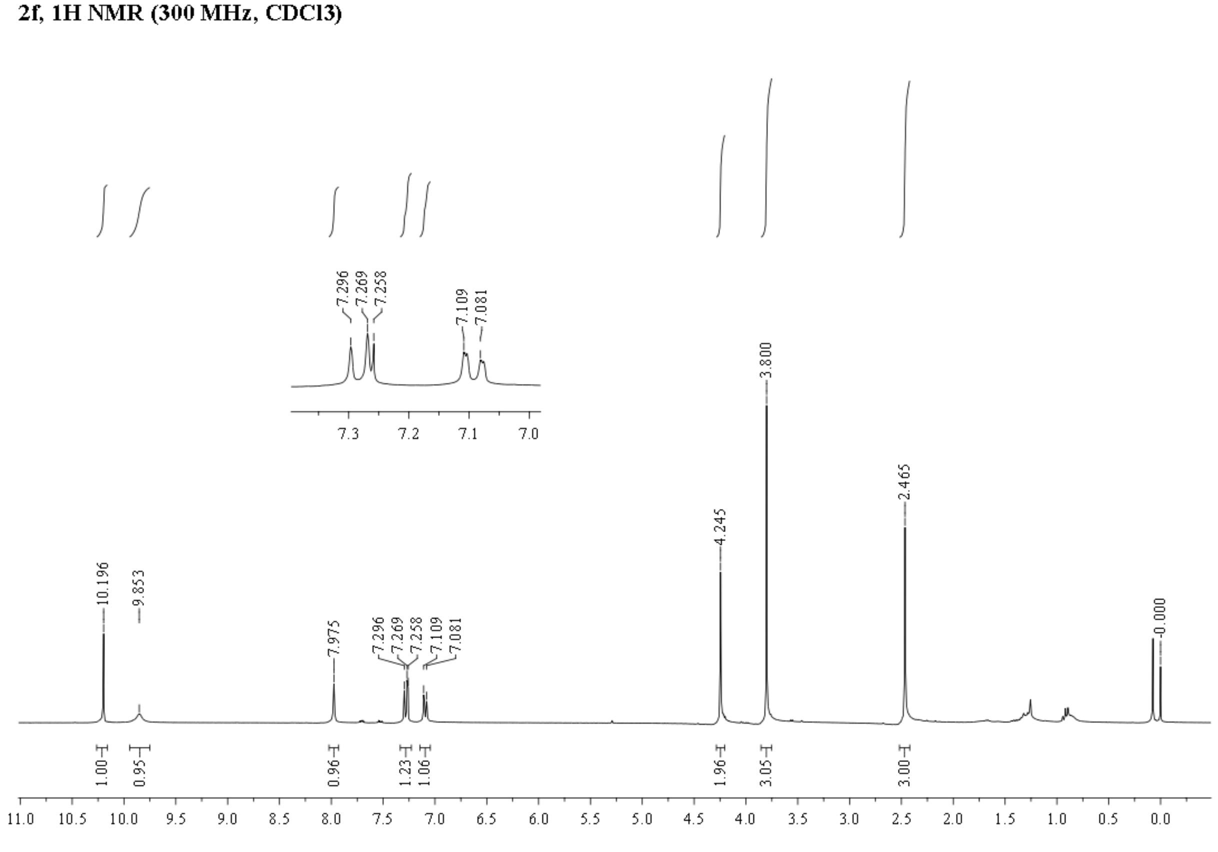

Figure S18. ^{13}C NMR spectrum of methyl 2-(3-formyl-5-methyl-1*H*-indol-2-yl)acetate (**2f**).

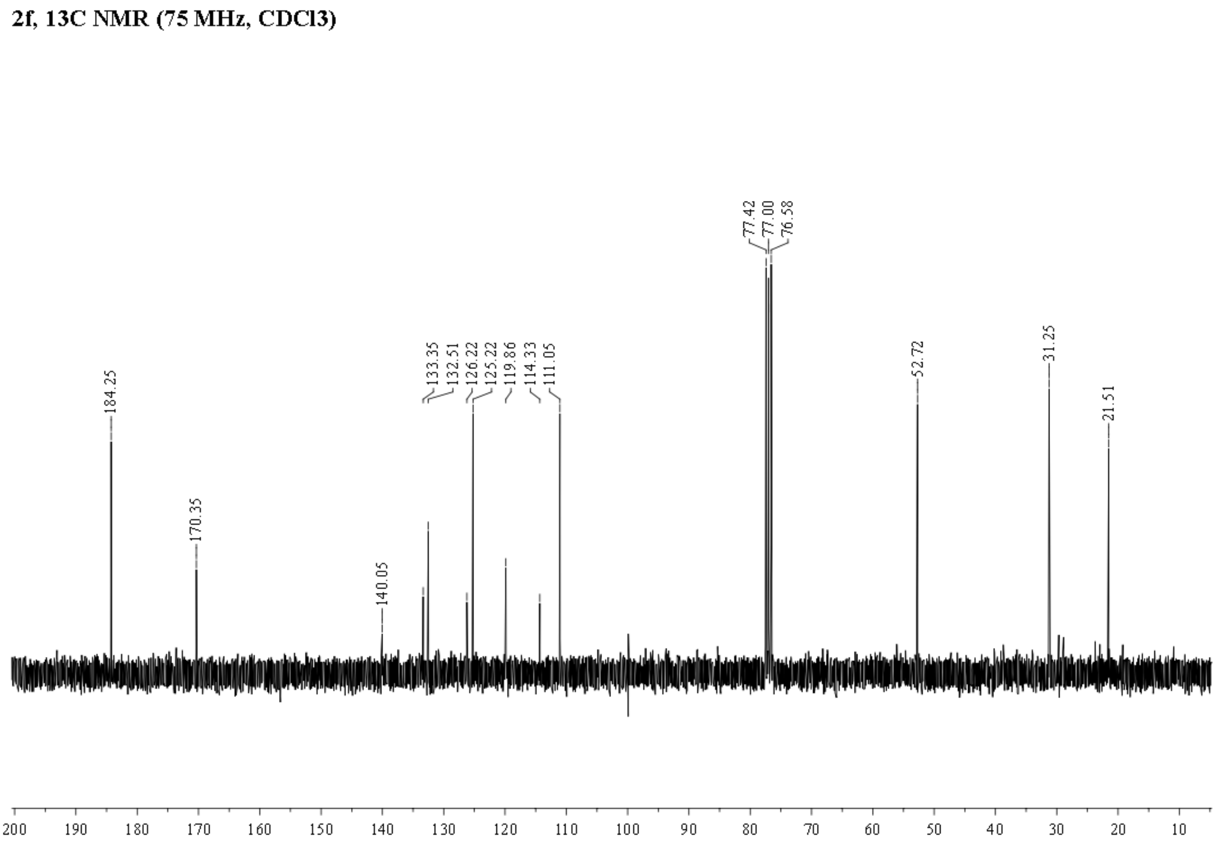

Figure S19. ^{1}H NMR spectrum of 5-methyl-1*H*-indole-3-carbaldehyde (**4f**).

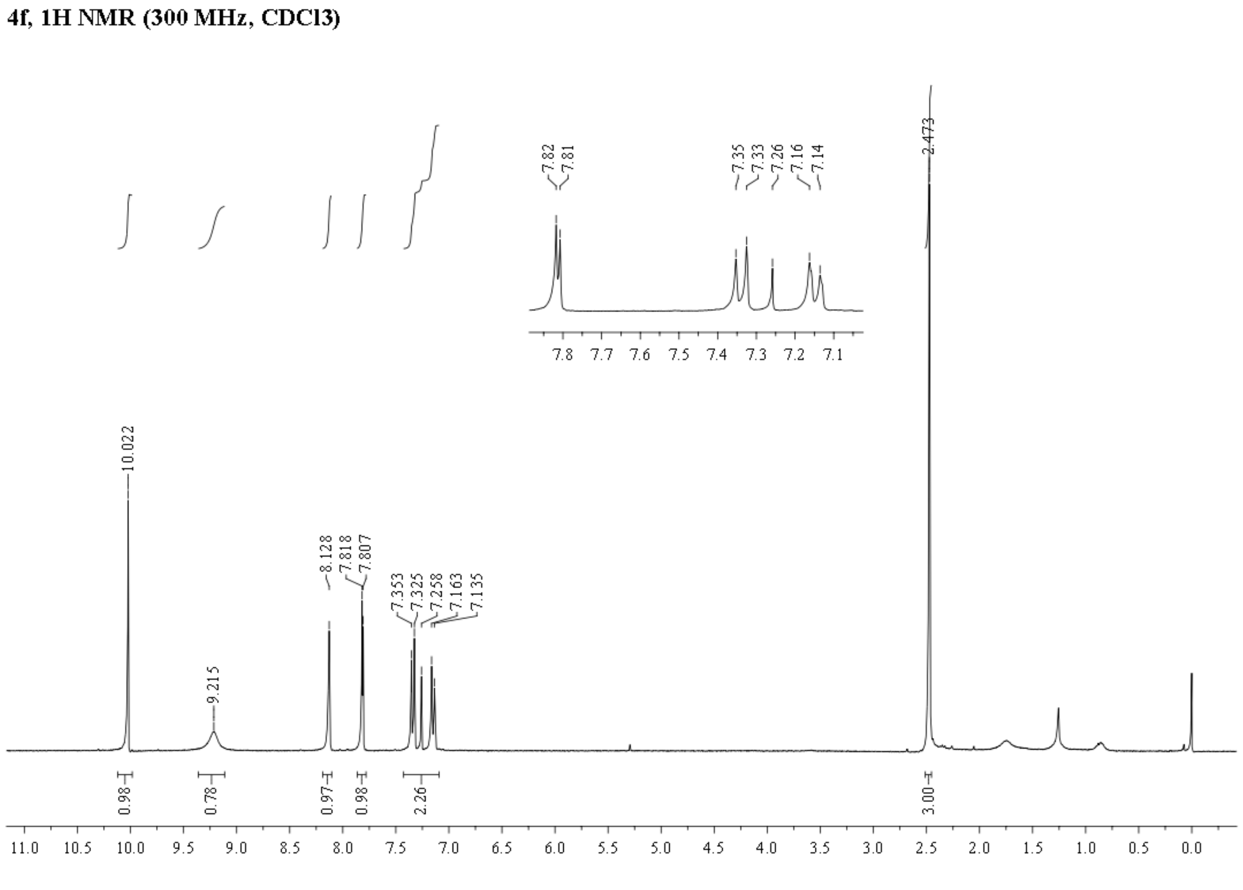

Figure S20. ^{1}H NMR spectrum of diethyl 2-(ethoxycarbonothioylthio)malonate (**3**).

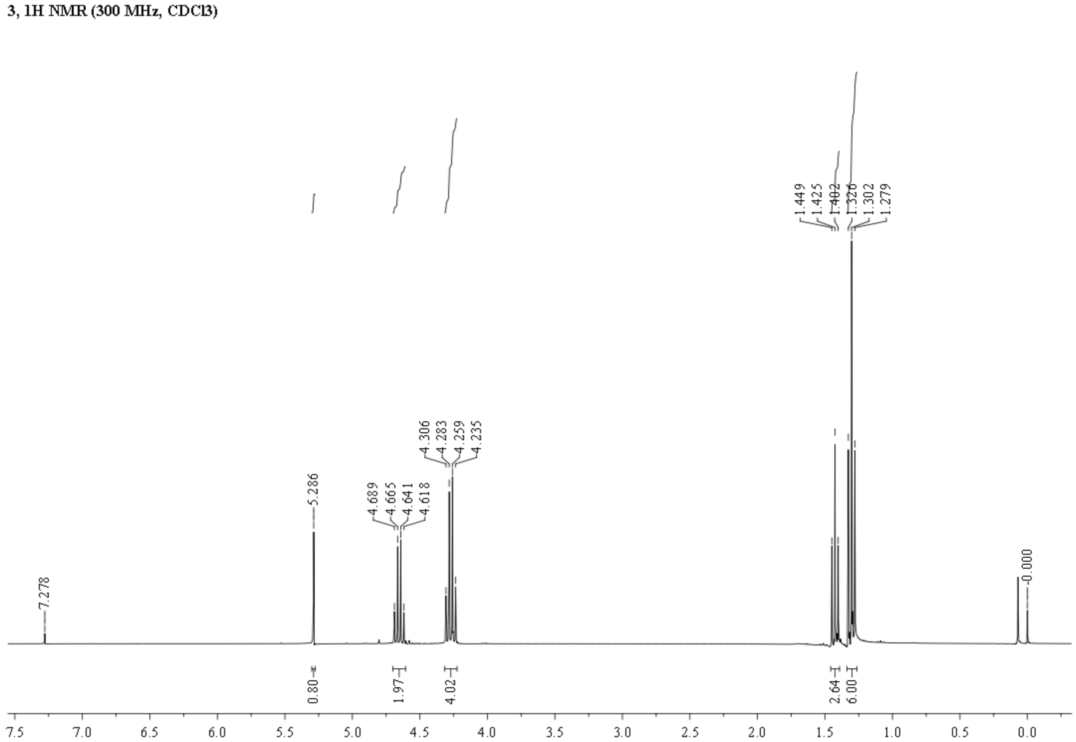

Figure S21. ^{13}C NMR spectrum of diethyl 2-(ethoxycarbonothioylthio)malonate (**3**).

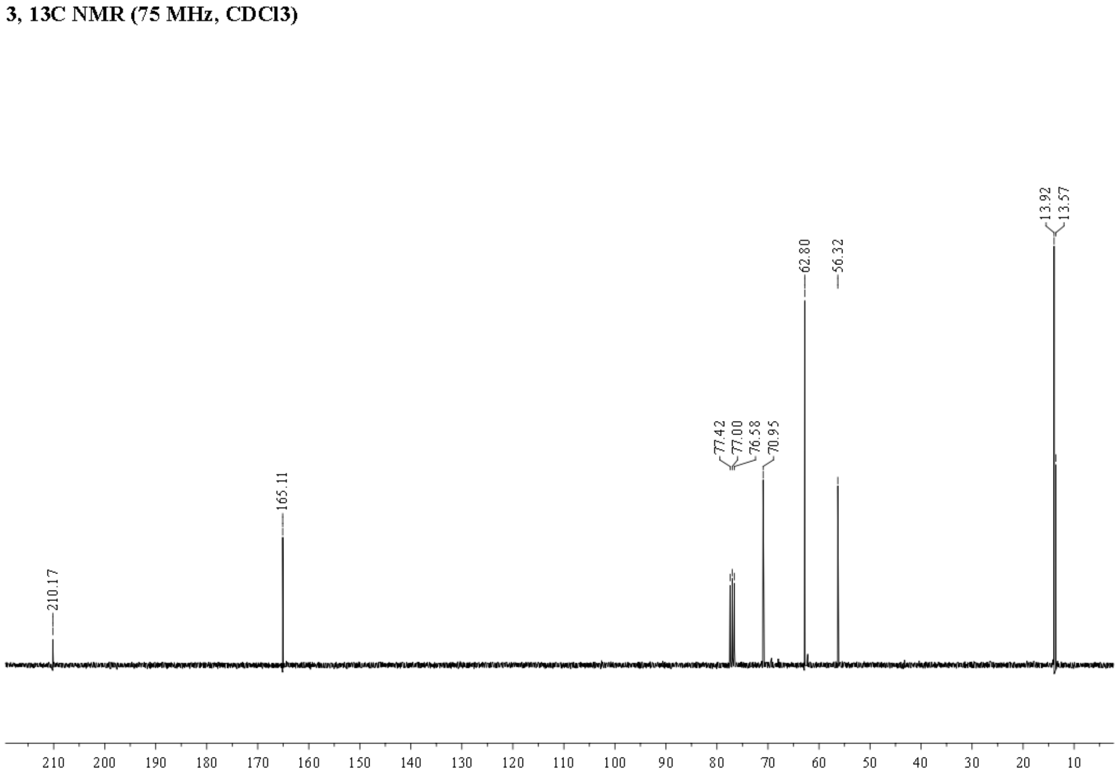

© 2014 by the authors; licensee MDPI, Basel, Switzerland. This article is an open access article distributed under the terms and conditions of the Creative Commons Attribution license (http://creativecommons.org/licenses/by/3.0/).
